# Supplementary material for: Predicting viral sensitivity to antibodies using genetic sequences and antibody similarities
Source: PLoS Comput Biol. 2026 Mar 23;22(3):e1014095. doi: 10.1371/journal.pcbi.1014095 (PMC13020759; doi:10.1371/journal.pcbi.1014095)
Supplement: S1 Text — (PDF) [file pcbi.1014095.s001.pdf]

Methods

Dimensional-reduction of viral sequences

To transform genetic sequences into an amenable data structure, we first process them into one-hot encoded sequences. For example, consider an aligned amino acid sequence $\tilde{\boldsymbol{g}} \in \mathcal{A}^{\tilde{L}}$, where $\mathcal{A} = \{\text{A, C, \ldots, Y, -}\}$ is the set of amino acids, including the alignment gap symbol '-', and $\tilde{L}$ is the length of the amino acid sequence. To convert this amino acid sequence $\tilde{\boldsymbol{g}}$ into a numerical form, we encode each amino acid as a $q = 21$-dimensional vector (corresponding to all possible amino acids and the gap symbol), consisting of zeros except for a single one at the position encoding the amino acid. By concatenating these q-dimensional column vectors $\tilde{L}$ times, we obtain the one-hot encoded sequence, which consists of $q\tilde{L}$ elements. Since the vast majority of elements in the one-hot encoded sequence are conserved across all considered viral sequences, we discard these conserved sites and retain only the variable sites. This results in $\boldsymbol{g} \in \{0,1\}^L$, a one-hot encoded sequence that contains only the variable sites, where L represents the number of variable sites in the one-hot sequence ensemble.

L, the number of variable sites of one-hot encoded sequences (approximately 7,000), is much larger than the number of neutralization values available for most antibodies (ranging from 2 to about 1,000). To learn a meaningful model while avoiding overfitting, we first project the one-hot encoded sequences into a lower-dimensional feature space. Formally, we consider a linear transformation of the one-hot encoded sequence:

$$\boldsymbol{x} = \Xi \boldsymbol{g}\,, \tag{3}$$

where $\Xi \in \mathbb{R}^{d \times L}$ is a projection matrix that maps the one-hot encoded sequences to the feature space. Each row of Ξ corresponds to a single feature and $\boldsymbol{x}$ represents the projected sequence (i.e., consisting of the d features of the single viral sequence), which will then be used to predict neutralization values.

In this study, we used the eigenmodes of the covariance matrix of genetic sequences as the Ξ matrix. Specifically, we obtained the covariance matrix as $C = \langle \boldsymbol{g}\boldsymbol{g}^\top \rangle - \langle \boldsymbol{g} \rangle \langle \boldsymbol{g} \rangle^\top$, where $\langle \cdot \rangle$ denotes the average over the viral genetic sequences in the data set. We then computed the eigenvectors $\boldsymbol{\xi}_i$ and eigenvalues λ_i of C. One can then write $C = -\sum_i^L \lambda_i \boldsymbol{\xi}_i \boldsymbol{\xi}_i^\top$, where $\boldsymbol{\xi}_i$ and λ_i are the i-th eigenvector and eigenvalue, respectively, with $\lambda_i \leq \lambda_{i+1} \leq 0$ for $i \in \{1, \ldots, L-1\}$. Finally, we constructed the Ξ matrix by selecting the first d eigenvectors as the row vectors of Ξ, such that the j-th element of i-th eigenvector $\Xi_{ij} = (\boldsymbol{\xi}_i)_j$.

To learn the neutralization values from the projected sequences $\boldsymbol{x}$, we set d based on the number of observed neutralization values. Specifically, we define $|\mathcal{N}_\mu|$ as the number of observed neutralization values and set:

$$d = \begin{cases} \lfloor |\mathcal{N}_\mu|/2 \rfloor\,, & \text{if } |\mathcal{N}_\mu| < 2d_{\max} \\ d_{\max}, & \text{otherwise} \end{cases}\,, \tag{4}$$

where $\lfloor a \rfloor$ represents the greatest integer that is equal to or smaller than a, and $d_{\max}$ denotes the maximum dimension of the feature vector, which we set as $d_{\max} = 100$ throughout. As we will discuss later, in the case of the grouped learning method, we replace $\mathcal{N}_\mu$ with $\mathcal{N}_k$, the union of all antibodies $\mathcal{N}_\mu$, where μ belongs to the k-th antibody group, such that $\mathcal{N}_k = \cup_{\mu \in k\text{-th antibody group}} \mathcal{N}_\mu$ The choice of the Ξ matrix, which defines the low-dimensional space, is flexible. As long as the proposed low-dimensional space effectively captures key features of the virus sequence ensemble, any appropriate matrix can be used. For example, non-negative matrix factorization (NMF) or other low-dimensional spaces that learn interpretable features such as structural or evolutionary signatures[51–53], could be applied for this purpose.

Neutralization similarities and grouping of antibodies

We consider the following antibody similarity matrix based on partially observed neutralization measurements. Let $(\boldsymbol{u}^*_\mu, \boldsymbol{u}^*_\nu)$ be a pair of neutralization vectors of antibodies μ and ν that are restricted to the $n_{\mu\nu}$ viruses where both antibodies have neutralization values (i.e., the intersection, $\mathcal{N}_\mu \cap \mathcal{N}_\nu$). The similarity matrix is then defined as

$$w_{\mu\nu} = \begin{cases} \frac{|\boldsymbol{u}^{*\top}_\mu \boldsymbol{u}^*_\nu|}{\|\boldsymbol{u}^*_\mu\| \|\boldsymbol{u}^*_\nu\|} & \mu \neq \nu \\ 0 & \mu = \nu \end{cases} \tag{5}$$

Here, $|\cdot|$ denotes the absolute value and $\|\cdot\|$ represents the L_2-norm (e.g., for a vector $\boldsymbol{v}$ with elements v_i, $\|\boldsymbol{v}\| = \left(\sum_i v_i^2\right)^{1/2}$). The first term represents the cosine or angular similarity between the two vectors in the restricted space, which ranges between zero and one.

The construction of the similarity matrix can vary, and may need to adjust based on types of data. For example, we considered scaling the weight matrix to downweight similarities when the number of shared entries is small, by introducing a scaling factor $(n_{\mu\nu}/N_V)^p$, where p is a small exponent $(0 < p \ll 1)$. However, we did not observe consistent improvements in prediction accuracy with this approach; therefore, we ultimately defined the similarity weights using cosine similarity alone.

As we will discuss in the next section, the similarity matrix ensures correlations between models that predict the neutralization activity of similar antibodies. The low-dimensional representation of the antibodies is also derived from the eigenmodes of the similarity matrix (**Fig. 1b**. Antibodies are grouped based on the similarity matrix, which is used to compute the graph Laplacian matrix for applying the spectral clustering algorithm[54].

Learning neutralization values

To predict the missing neutralization values, we learn the relationship between sequences and neutralization values. To do this, we define a model that reproduces the neutralization value as $u_{\mu\alpha} = f(\boldsymbol{x}_\alpha \mid \boldsymbol{\theta}_\mu) + \epsilon_{\mu\alpha}$. Here, $f : \mathbb{R}^d \to \mathbb{R}$ is a function, $\boldsymbol{\theta}_\mu$ is a model parameter that captures the neutralization values of antibody μ, and $\epsilon_{\mu\alpha}$ is the uncertainty, which we assume is normally distributed with a mean of zero. Let $\boldsymbol{\theta}$ and $\boldsymbol{x}$ be $\boldsymbol{\theta} = (\boldsymbol{\theta}_1^\top, \ldots, \boldsymbol{\theta}_{N_A}^\top)^\top$ and $\boldsymbol{x} = (\boldsymbol{x}_1^\top, \ldots, \boldsymbol{x}_{N_V}^\top)^\top$,

respectively. Using Bayesian rule, the optimal model parameters are obtained as a maximum a posterior estimator, given by

$$\hat{\boldsymbol{\theta}} = \arg\max_{\boldsymbol{\theta}} P(\boldsymbol{x} \mid \boldsymbol{\theta}) P(\boldsymbol{\theta} \mid \boldsymbol{\lambda}) , \quad \textbf{(6)}$$

where $\boldsymbol{\lambda} = (\lambda, \lambda')^\top$ is a set of parameters characterizing the distribution of $\boldsymbol{\theta}$, which we will discuss below.

Here, the likelihood function is given as

$$P(\boldsymbol{x} \mid \boldsymbol{\theta}) \propto \exp\left(-\frac{1}{2} \sum_{\mu \in \mathcal{N}_A} \sum_{\alpha \in \mathcal{N}_\mu} \left(\boldsymbol{x}_\alpha^\top \boldsymbol{\theta}_\mu - u_{\mu\alpha} \right)^2 \right) . \quad \textbf{(7)}$$

For the prior distribution, we assume that antibodies with similar neutralization profiles share similar model parameters. Formally, the standard deviation of the difference between these parameters is inversely correlated with the similarity weights, therefore we represent it as

$$P(\boldsymbol{\theta} \mid \boldsymbol{\lambda}) \propto \exp\left(-\frac{\lambda}{2} \|\boldsymbol{\theta}\|^2 - \frac{\lambda'}{2} \sum_{\mu, \nu \in \mathcal{N}_A} w_{\mu\nu} \|\boldsymbol{\theta}_\mu - \boldsymbol{\theta}_\nu\|^2 \right) . \quad \textbf{(8)}$$

The optimal parameter, derived from the logarithm of the maximization of a posterior distribution, is the solution to its stationary condition, expressed as

$$\boldsymbol{\partial}_{\boldsymbol{\theta}} \log \left(P(\boldsymbol{x} \mid \boldsymbol{\theta}) P(\boldsymbol{\theta} \mid \boldsymbol{\lambda}) \right) |_{\boldsymbol{\theta} = \hat{\boldsymbol{\theta}}} = \mathbf{0} \, ,. \quad \textbf{(9)}$$

To solve the stationary equation, let us define a matrix as

$$\mathcal{L}_{\mu\nu} = \begin{cases} \sum_\nu w_{\mu\nu} & \text{for } \mu = \nu \\ -w_{\mu\nu} & \text{for } \mu \neq \nu \, , \end{cases} \quad \textbf{(10)}$$

which is equivalent to the weighted graph Laplacian matrix[54], and its lifted matrix $\mathfrak{L}$ as

$$\mathfrak{L} := \mathcal{L} \otimes \mathrm{I}_d \, , \quad \textbf{(11)}$$

where $\otimes$ and I_d represent the outer product and d-dimensional identity matrix, respectively. Here, $\mathfrak{L}$ is a block diagonal matrix, with entries $\mathfrak{L}_{\mu\nu} \mathrm{I}_d$ in the block spanning row indices from $(\mu - 1) N_A + 1$ to μN_A and column indices from $(\nu - 1) N_A + 1$ to νN_A. Finally, the analytical expression of the optimal model parameter $\hat{\boldsymbol{\theta}}$ can be expressed as

$$\hat{\boldsymbol{\theta}} = (X + \lambda' \mathfrak{L})^{-1} \boldsymbol{\psi} \, , \quad \textbf{(12)}$$

with

$$\mathfrak{X} = \mathrm{diag}\left(\left(\sum_{\alpha \in \mathcal{N}_\mu} \boldsymbol{x}_\alpha \boldsymbol{x}_\alpha^\top + \lambda \mathrm{I}_d \right)_{\mu=1}^{N_A} \right) , \quad \textbf{(13)}$$

which is a block diagonal matrix consisting of N_A d-by-d blocks, and

$$\boldsymbol{\psi} = \begin{pmatrix} \sum_{\alpha \in \mathcal{N}_1} u_{1\alpha} \boldsymbol{x}_\alpha \\ \vdots \\ \sum_{\alpha \in \mathcal{N}_{N_A}} u_{N_A \alpha} \boldsymbol{x}_\alpha \end{pmatrix} . \quad \textbf{(14)}$$

In this construction, and obtained equations (Eq. (12) to Eq. (14)), when the $\mathcal{L}$ matrix is diagonal ($\mathcal{L}_{\mu\nu} = 0$ for $\mu \neq \nu$), that is, all the similarity weights are zero, resulting in the $\mathfrak{L}$ matrix becoming a trivial (block) diagonal matrix. In this case, Eq. (12) is independent for each antibody and recovers the naive linear regression solution. We refer to this as independent neutralization learning (INL) method. However, as long as the elements of $\mathcal{L}_{\mu\nu} \neq 0$, the equations for antibodies μ and ν become dependent, and their influences are interconnected. In particular, a group of antibodies with similar higher weight values influences each other, and we refer to this as the grouped neutralization learning (GNL) method.

Efficient computation for grouped learning methods.

As discussed above, the similarity weight can be used to identify distinct antibody groups by applying a standard spectral clustering algorithm[54]. Once antibodies are grouped by K groups (where K is a positive integer and a hyperparameter) based on similar neutralization profiles, the equation to be solved (Eq. (12)) is reduced to smaller sub-problems: assuming that the weight matrix involves K disjoint blocks, then the full Laplacian matrix is divided into K sub-Laplacian matrices, $\mathfrak{L} = \mathrm{diag}((\mathfrak{L}^k)_{k=1}^K)$, where $\mathfrak{L}^k$ is k-th sub-Laplacian matrix. Therefore, the original problem Eq. (12) is reduced to solve the following sub-problem for each k:

$$(\mathfrak{X}^k + \lambda' \mathfrak{L}^k) \boldsymbol{\theta}^k - \boldsymbol{\psi}^k = \mathbf{0} \, . \quad \textbf{(15)}$$

Here $\mathfrak{X}^k, \mathfrak{L}^k, \boldsymbol{\theta}^k$, and $\boldsymbol{\psi}^k$ are ones that associated with the k-th group.

This divided rule efficiently reduces computational complexity, especially when similar antibodies are classified into smaller groups. This divided rule allows us to employ adaptive group-dependent regularization strength λ'_k, and group-dependent dimension d_k, which is given by $d_k = \lfloor |\mathcal{N}_k|/2 \rfloor$ if $|\mathcal{N}_k| < 2 d_{\max}$ other wise $d_k = d_{\max}$, where $\mathcal{N}_k$ represents the union of $\mathcal{N}_\mu$ for all antibodies μ belong to the k-th antibody group.

Iterative matrix inversion algorithm The matrices $\mathfrak{L}$ and $\mathfrak{X}$ are individually invertible with low computational costs, as $\mathfrak{L}^{-1} = \mathcal{L}^{-1} \otimes \mathrm{I}_d$, and the inversion of a block diagonal matrix is computationally simpler. However, combining $\mathfrak{L}$ and $\mathfrak{X}$ disrupts these advantageous properties. Therefore, inverting the combination of them could take a longer time. To accelerate the process, we could employ an iterative algorithm to obtain the solution.

The Laplacian matrix $\mathcal{L}$ should have at least one singular mode, as $\mathcal{L}\mathbf{1} = \mathbf{0}$, with $\mathbf{1}$ being the d-dimensional vector whose elements are all 1. If antibodies are divided into K distinct and disjoint classes, then the rank of the Laplacian matrix is $r = N_A - K$. We denote the Laplacian matrix as $\mathcal{L} = \sum_{l=1}^r \boldsymbol{\rho}_l \boldsymbol{\rho}_l^\top$, where $\boldsymbol{\rho}_l$ is the l-th eigenvector corresponding to strictly positive eigenvalues. The extended Laplacian matrix $\mathfrak{L}$ is then represented as follows:

$$\mathfrak{L} = \sum_{l=1}^{rd} \tilde{\boldsymbol{\rho}}_l \tilde{\boldsymbol{\rho}}_l^\top , \quad \textbf{(16)}$$

with

$$\tilde{\boldsymbol{\rho}}_l = \boldsymbol{\rho}_l \otimes \boldsymbol{e}_i \ , \tag{17}$$

where $\boldsymbol{e}_i$ has one at the i-th element and all other elements are zero. Based on the efficient computation algorithm for obtaining the inverse of the sum of matrices[55], the inverse of the matrices can be solved iteratively by:

$$\begin{aligned} G_{l+1}^{-1} &= G_l^{-1} - g_l G_l^{-1} \tilde{\boldsymbol{\rho}}_l \left(G_l^{-1} \tilde{\boldsymbol{\rho}}_l \right)^\top \\ G_1^{-1} &= \mathfrak{X}^{-1} \\ g_l &= 1 / \left(1 + \tilde{\boldsymbol{\rho}}_l^\top G_l^{-1} \tilde{\boldsymbol{\rho}}_l \right) \ . \end{aligned} \tag{18}$$

By iteratively solving G_l^{-1} up to $l = rd$ times, we get $G_{rd}^{-1} = (\mathfrak{X} + \lambda' \mathfrak{L})^{-1}$.

The naive matrix-vector multiplication for $G_l^{-1} \tilde{\boldsymbol{\rho}}_l$ requires $\mathcal{O}(r^2 d^2)$ operations; however $\tilde{\boldsymbol{\rho}}_l$ is a sparse matrix and it requires only $\mathcal{O}(r^2)$ operations, the outer-product also takes solely $\mathcal{O}(r^2)$ operations, and requires updates only $\mathcal{O}(r^2)$ elements at each l-th iteration. Therefore, after repeating the operation $\mathcal{O}(rd)$ times, the total computational cost is only $\mathcal{O}(r^3 d)$, which is much smaller than $\mathcal{O}(r^3 d^3)$ of the naive inverse of the matrix.

Calculation of effective weight values for the specific mutations

The proposed model directly links viral genetic sequences to viral sensitivity values. Let $\boldsymbol{\theta}_\mu$ denote the model parameters for antibody μ, and let $\boldsymbol{x}_\alpha$ denote the projected sequence representation of viral strain α. The viral sensitivity of strain α to antibody μ is then given by

$$u_{\mu\alpha} = \boldsymbol{\theta}_\mu^\top \boldsymbol{x}_\alpha \, .$$

In this study, this expression can be written as a linear combination over individual mutations encoded in the one-hot genetic sequence $\boldsymbol{g}_\alpha \in \{0,1\}^L$.

$$u_{\mu\alpha} = \sum_i^L b_{\mu,i} g_{\alpha,i} \, . \tag{19}$$

Here, $b_{\mu,i}$ represents the weight, or contribution, of mutation i to the sensitivity of antibody μ. These weights can be expressed as

$$b_{\mu,i} = \sum_i^L \left(\Xi^\top \boldsymbol{\theta}_\mu \right)_i g_{\alpha,i} \, . \tag{20}$$

where Ξ denotes the projection matrix mapping model parameters to mutation space. By definition, $\boldsymbol{g}_\alpha$ is a one-hot–encoded vector, so only mutations present in strain α (i.e., entries with nonzero values) contribute to the viral sensitivity $u_{\mu\alpha}$. Although our analysis is based on a linear model, the underlying framework can be naturally extended to more complex models, including those that explicitly incorporate epistatic interactions.

Processing the neutralization data

We retrieved IC50 neutralization values from the CATNAP database provided by HIV sequence database at Los Alamos National Laboratory (LANL)[56]. By November 2024, over 172,000 IC50 neutralization values were available, and more than 159,000 of these values were tested with one of 806 monoclonal antibodies and 2,486 viral strains. Below, we describe the data processing steps, which are largely consistent with those used in Einav et al.

Some IC50 values exceeded experimental thresholds, typically above 50 μg/mL or below 0.01 μg/mL. To address this, we replaced values above the threshold with double the threshold and those below it with half the threshold (e.g., values >50 μg/mL were set to 100 μg/mL, and values <0.01 μg/mL were set to 0.005 μg/mL). Since the saturated IC50 values can vary across antibodies, we tested the effect of imposing a fixed ceiling of 50 μg/mL. However, this approach consistently resulted in lower accuracy across methods. Therefore, we retained neutralization values as given in each data set. After this adjustment, we transformed the raw IC50 values using the logarithm. This transformation aligned the scale with viral fitness under antibody pressure and emphasized smaller neutralization values (same as the prior work[57]). We then identified unique antibodies and virus strains, arranging the neutralization values into a matrix with rows corresponding to antibodies and columns corresponding to viruses. For antibody-virus pairs with multiple neutralization values, we computed the arithmetic average of the (log-transformed) values. All sources of experimental data were treated equally (same as ref.[57]). Although we haven't applied it due to the limited data, normalization of neutralization values could potentially reduce bias between experiments if sufficient values for certain antibody-virus pairs are available across multiple experiments. At this stage, we obtain $N_A = 806$ antibodies and 2,486 viruses, resulting in 90,738 neutralization values (about 4.5% of the total matrix.) We limited our analysis to virus strains with available envelope protein sequences, ultimately selecting 2,229 strains. Additionally, to improve data quality, we excluded virus strains with fewer than 10 neutralization values, resulting in $N_V = 1,147$ strains. After filtering out antibodies without neutralization values (none of the antibodies were the case, so $N_A = 806$), we were left with 81,193 neutralization values, resulting 4.5% of the filtered neutralization matrix.

For the intrahost HIV neutralization data, we retrieved the neutralization values from Gao et al.[58]. As noted in the main section, the neutralization matrix consists of $N_A = 22$ antibodies, $N_V = 109$ viruses, and a total of 1,723 neutralization values.

Processing the viral sequences

In this study, we focused on the Env gene, which encodes the virus envelope protein targeted by antibodies. Using the alignment software provided by LANL[59], we aligned the viral Env protein sequences to the HIV reference strain HXB2. This allowed us to map amino acid positions to standardized indices commonly used in HIV research. To simplify anal-

ysis, we only considered positions that were not alignment gaps in the HXB2 sequence. This resulted in 856 positions that were present in most viral sequences. We converted the aligned viral Env sequences into one-hot encoded vectors. Each position (21 possibilities: 20 amino acids plus a gap) was represented as a binary vector with a single ones corresponding to the observed allele and zero elsewhere. This produced $856 \times 21 = 17,976$ binary features per sequence. Since many positions were conserved across sequences, we retained only variable positions, yielding $L = 7,802$ variable positions. The resulting one-hot encoded sequences still contain a large number of variables. Further dimensional reduction is described in the following section.

Details of conditions for genotype-neutralization learning

The number of principal components used depended on the number of observed neutralization values. We set the minimum number of components to 2 and the maximum to 100, choosing half the number of observations for most cases. The strength of the L_2 regularization on the parameter was fixed at $\lambda = 5$, and the regularization for the similarity of the model was fixed at $\lambda' = 1$ for all simulations.

Low-rank approximation

As detailed in the main text, we used standard singular value decomposition (SVD) instead of the robust principal component analysis (rPCA) method employed in previous studies by Einav et al.[57]. Our results showed no significant differences between SVD and rPCA.

Before applying the low-rank approximation, missing values were completed using the GNL model based on viral sequences. For the Einav et al. method, missing values were replaced by mean estimates calculated from observed data for the corresponding antibodies and viruses. Mean values were subtracted before performing low-rank approximation and added back afterward, following procedures from the Einav et al. study[57].

The optimal rank value is determined as the minimum rank value where the variance explanation exceeds 95%, as per the optimization scheme of the Einav et al. method[57]. The variance explanation for the matrix rank ρ is defined as the cumulative sum of the squared eigenvalues, $\sum_{i=1}^{\rho} \sigma_i^2 / \sum_{i=1}^{\rho_{\max}} \sigma_i^2$. Here, σ_i is the i-th singular value ($\sigma_i \leq \sigma_{i+1}$), and $\rho_{\max}$ is the total number of singular modes.

The accuracy of neutralization imputation depends on the matrix rank. In **Fig. 1**e and **Supplementary Fig. 3**, we show how accuracy varies with matrix rank ρ: the accuracy changes significantly with rank in the method by Einav et al., while the GNL method is more robust and generally achieves higher accuracy. **Supplementary Fig. 1** also compares the variance explanation profiles and highlights the optimal rank values.

Code availability

Sets of data and computer codes available in the GitHub repository: https://github.com/bartonlab/paper-ic50-prediction . The repository contains neutralization datasets and HIV-1 envelop genetic sequences. The most recent neutralization data and HIV viral sequences datasets are obtained from the CATNAP (http://hiv.lanl.gov/catnap) and LANL (https://www.hiv.lanl.gov) databases, respectively.

References

51. Cocco, S., Monasson, R. & Weigt, M. From principal component to direct coupling analysis of coevolution in proteins: Low-eigenvalue modes are needed for structure prediction. *PLoS computational biology* **9**, e1003176 (2013).
52. Tubiana, J., Cocco, S. & Monasson, R. Learning protein constitutive motifs from sequence data. *Elife* **8**, e39397 (2019).
53. Shimagaki, K. & Weigt, M. Selection of sequence motifs and generative hopfield-potts models for protein families. *Physical Review E* **100**, 032128 (2019).
54. Von Luxburg, U. A tutorial on spectral clustering. *Statistics and computing* **17**, 395–416 (2007).
55. Miller, K. S. On the inverse of the sum of matrices. *Mathematics magazine* **54**, 67–72 (1981).
56. Los Alamos National Laboratory. HIV sequence database (2023). URL https://www.hiv.lanl.gov. Accessed: 703010505 and 703010848 in patient code.
57. Einav, T. & Cleary, B. Extrapolating missing antibody-virus measurements across serological studies. *Cell Systems* **13**, 561–573 (2022).
58. Gao, F. *et al.* Cooperation of b cell lineages in induction of HIV-1-broadly neutralizing antibodies. *Cell* **158**, 481–491 (2014).
59. Los Alamos National Laboratory. HIVAlign: HIV sequence alignment tool (2023). URL https://www.hiv.lanl.gov/content/sequence/VIRALIGN/viralign.html.
60. Julien, J.-P. *et al.* Broadly neutralizing antibody PGT121 allosterically modulates CD4 binding via recognition of the HIV-1 gp120 V3 base and multiple surrounding glycans. *PLoS pathogens* **9**, e1003342 (2013).
61. Garces, F. *et al.* Structural evolution of glycan recognition by a family of potent HIV antibodies. *Cell* **159**, 69–79 (2014).
62. Anthony, C. *et al.* Cooperation between strain-specific and broadly neutralizing responses limited viral escape and prolonged the exposure of the broadly neutralizing epitope. *Journal of virology* **91**, 10–1128 (2017).
63. Orwenyo, J. *et al.* Systematic synthesis and binding study of HIV V3 glycopeptides reveal the fine epitopes of several broadly neutralizing antibodies. *ACS chemical biology* **12**, 1566–1575 (2017).
64. Bricault, C. A. *et al.* HIV-1 neutralizing antibody signatures and application to epitope-targeted vaccine design. *Cell host & microbe* **25**, 59–72 (2019).
65. Stephenson, K. E. *et al.* Safety, pharmacokinetics and antiviral activity of PGT121, a broadly neutralizing monoclonal antibody against HIV-1: a randomized, placebo-controlled, phase 1 clinical trial. *Nature medicine* **27**, 1718–1724 (2021).
66. Jeffy, J. *et al.* Alternative substitutions of N332 in HIV-1AD8 gp120 differentially affect envelope glycoprotein function and viral sensitivity to broadly neutralizing antibodies targeting the V3-glycan. *Mbio* **15**, e02686–23 (2024).
67. Yoon, H. *et al.* CATNAP: a tool to compile, analyze and tally neutralizing antibody panels. *Nucleic acids research* **43**, W213–W219 (2015).
68. Liao, H.-X. *et al.* Co-evolution of a broadly neutralizing HIV-1 antibody and founder virus. *Nature* **496**, 469–476 (2013).
69. Guzzo, C. *et al.* Structural constraints at the trimer apex stabilize the HIV-1 envelope in a closed, antibody-protected conformation. *Mbio* **9**, 10–1128 (2018).
70. Zacharopoulou, P. *et al.* Prevalence of resistance-associated viral variants to the hiv-specific broadly neutralising antibody 10-1074 in a UK bNAb-naïve population. *Frontiers in Immunology* **15**, 1352123 (2024).
71. Chuang, G.-Y. *et al.* Structural survey of broadly neutralizing antibodies targeting the HIV-1 Env trimer delineates epitope categories and characteristics of recognition. *Structure* **27**, 196–206 (2019).
72. Martinez, D. *et al.* Maternal broadly neutralizing antibodies can select for neutralization-resistant, infant-transmitted/founder HIV variants. mBio 11: e00176-20 (2020).
73. Radford, C. E. & Bloom, J. D. Comprehensive maps of escape mutations from antibodies 10-1074 and 3BNC117 for Envs from two divergent HIV strains. *Journal of Virology* **99**, e00195–25 (2025).
74. Cohen, P. *et al.* Resistance mutations that distinguish HIV-1 envelopes with discordant VRC01 phenotypes from multi-lineage infections in the HVTN703/HPTN081 trial: implications for cross-resistance. *Journal of Virology* **99**, e01730–24 (2025).
75. Gieselmann, L. *et al.* Profiling of HIV-1 elite neutralizer cohort reveals a CD4bs bnAb for HIV-1 prevention and therapy. *Nature Immunology* 1–14 (2025).
76. Cale, E. M. *et al.* Virus-like particles identify an HIV V1V2 apex-binding neutralizing antibody that lacks a protruding loop. *Immunity* **46**, 777–791 (2017).
77. van Dorsten, R. T. *et al.* Neutralization breadth and potency of single-chain variable fragments derived from broadly neutralizing antibodies targeting multiple epitopes on the HIV-1 envelope. *Journal of Virology* **94**, 10–1128 (2020).

78. Wang, W. *et al.* N463 glycosylation site on v5 loop of a mutant gp120 regulates the sensitivity of HIV-1 to neutralizing monoclonal antibodies VRC01/03. *JAIDS Journal of Acquired Immune Deficiency Syndromes* **69**, 270–277 (2015).
79. Brunel, F. M. *et al.* Structure-function analysis of the epitope for 4E10, a broadly neutralizing human immunodeficiency virus type 1 antibody. *Journal of virology* **80**, 1680–1687 (2006).
80. Banerjee, S. *et al.* Evaluation of a novel multi-immunogen vaccine strategy for targeting 4E10/10E8 neutralizing epitopes on HIV-1 gp41 membrane proximal external region. *Virology* **505**, 113–126 (2017).
81. Yuan, M. *et al.* Conformational plasticity in the HIV-1 fusion peptide facilitates recognition by broadly neutralizing antibodies. *Cell Host & Microbe* **25**, 873–883 (2019).
82. Kong, R. *et al.* Fusion peptide of HIV-1 as a site of vulnerability to neutralizing antibody. *Science* **352**, 828–833 (2016).
83. Dingens, A. S. *et al.* Complete functional mapping of infection-and vaccine-elicited antibodies against the fusion peptide of HIV. *PLoS pathogens* **14**, e1007159 (2018).
84. Williamson, B. D. *et al.* Super learner prediction of NAb panels (SLAPNAP): a containerized tool for predicting combination monoclonal broadly neutralizing antibody sensitivity. *Bioinformatics* **37**, 4187–4192 (2021).
85. Bonsignori, M. *et al.* Maturation pathway from germline to broad hiv-1 neutralizer of a cd4-mimic antibody. *Cell* **165**, 449–463 (2016).
86. Saunders, K. O. *et al.* Targeted selection of hiv-specific antibody mutations by engineering b cell maturation. *Science* **366**, eaay7199 (2019).
87. Kreer, C. *et al.* Probabilities of developing hiv-1 bnab sequence features in uninfected and chronically infected individuals. *Nature communications* **14**, 7137 (2023).
