# Supplementary material for: Predicting viral sensitivity to antibodies using genetic sequences and antibody similarities
Source: PLoS Comput Biol. 2026 Mar 23;22(3):e1014095. doi: 10.1371/journal.pcbi.1014095 (PMC13020759; doi:10.1371/journal.pcbi.1014095)
Supplement: S1 Table — (PDF) [file pcbi.1014095.s002.pdf]

Data name and type	Descriptions
CATNAP (Neutralization data) [1]	This dataset consists of more than 166,000 IC50 values, over 76,000 IC80 values, and approximately 4,000 ID50 values. In total, more than 73,000 virus–antibody pairs have both IC50 and IC80 measurements.
CATNAP (HIV-1 Env sequence data) [1, 2]	A total of 2,716 HIV-1 Env sequences are included. Sequences incorporate a PNGS-specific symbol ('O') to explicitly represent potential N-linked glycosylation sites.
CATNAP (Alignment-free features) [3, 1]	For each of the 2,716 Env sequences, alignment-free features were extracted from variable regions V1–V5 and the combined V1+V2 region. These features include counts of PNGSs (matching N-X-S or N-X-T, with X $\neq$ P), non-glycosylated motifs (matching N-P-S or N-P-T), tandem or overlapping PNGSs (e.g., N-N-S-T or N-N-T-S), net charge, and loop length.
CH505 (Intrahost neutralization data) [4]	This dataset includes neutralization measurements for two CD4 binding site (CD4bs) bnAb lineages: CH103 and CH235. The CH103 dataset consists of 124 viral sequences tested against 13 antibodies, while the CH235 dataset includes 41 sequences tested against 10 antibodies.
CH505 (Longitudinal Env sequence data) [5]	Longitudinal Env sequences were collected from a single host, comprising 624 sequences sampled at 23 time points ranging from 4 to 323 weeks post-infection.

S1 Table Summary of the data sources used in this study.

References

[1] Hyejin Yoon et al. "CATNAP: a tool to compile, analyze and tally neutralizing antibody panels". In: *Nucleic acids research* 43.W1 (2015), W213–W219.

[2] Los Alamos National Laboratory. *HIV Sequence Database*. Accessed: 703010505 and 703010848 in patient code. 2023. URL: https://www.hiv.lanl.gov.

[3] Christine A Bricault et al. "HIV-1 neutralizing antibody signatures and application to epitope-targeted vaccine design". In: *Cell host & microbe* 25.1 (2019), pp. 59–72.

[4] Feng Gao et al. "Cooperation of B cell lineages in induction of HIV-1-broadly neutralizing antibodies". In: *Cell* 158.3 (2014), pp. 481–491.

[5] Hua-Xin Liao et al. "Co-evolution of a broadly neutralizing HIV-1 antibody and founder virus". In: *Nature* 496.7446 (2013), pp. 469–476.
