# Supplementary material for: Predicting viral sensitivity to antibodies using genetic sequences and antibody similarities
Source: PLoS Comput Biol. 2026 Mar 23;22(3):e1014095. doi: 10.1371/journal.pcbi.1014095 (PMC13020759; doi:10.1371/journal.pcbi.1014095)
Supplement: S2 Table — Summary of key parameters, their meaning, suggested values, and guidelines for adjustment based on data characteristics. (PDF) [file pcbi.1014095.s003.pdf]

| Notation | Meaning | Value or how to adjust it |
|---|---|---|
| $\rho$ | Optimal neutralization matrix rank | Optimized as the minimum integer $\rho$ satisfying $\sum_{i=1}^{\rho} \sigma_i^2 \Big/ \sum_{i=1}^{\rho_{\max}} \sigma_i^2 > 0.95$, where $\sigma_i$ is the $i$-th singular value of the neutralization matrix. |
| $K$ | Maximum number of antibody groups | Use $K = 100$ for CATNAP. Adjusted to ensure well-distributed clusters while maintaining similarity within each antibody group. Results were insensitive in the range $K \in [50, 200]$ . |
| $K$ | Max antibody groups (Intrahost) | Use $K = 5$ for intrahost neutralization data. |
| $d_{\max}$ | Max dimensions of projected sequences | Should be based on the maximum number of neutralization values across all antibodies. |
| $d$ | Dimensions of projected sequences | Optimized based on the number of viruses for which at least one antibody in each group has a neutralization value. |
| $d_k$ | Antibody group–specific dimension | Can be optimized depending on variation in neutralization profiles within each antibody group. |
| $\lambda$ | Regularization on regression parameters | We use $\lambda = 10$ for all conditions. Results were insensitive to values in the range $\lambda \in [5, 30]$. |
| $\lambda'$ | Similarity regularization between model parameters $\boldsymbol{\theta}_\mu$ and $\boldsymbol{\theta}_\nu$ | We use $\lambda' = 5$. This value was also insensitive in the range $\lambda' \in [1, 20]$ . Note that $\lambda$ and $\lambda'$ should be jointly tuned. |

**S2 Table Model hyperparameters.** Summary of key parameters, their meaning, suggested values, and guidelines for adjustment based on data characteristics.
