# Supplementary material for: Predicting viral sensitivity to antibodies using genetic sequences and antibody similarities
Source: PLoS Comput Biol. 2026 Mar 23;22(3):e1014095. doi: 10.1371/journal.pcbi.1014095 (PMC13020759; doi:10.1371/journal.pcbi.1014095)
Supplement: S3 Table — This table summarizes key mutations identified based on their contributions to the predicted log IC50 values. Mutations are selected from the top 0.05% according to the absolute values of their weight parameters, which quantify their contributions to the predicted log IC50, across all antibodies within the same antibody class. For these top-ranked mutations, the table reports the average contribution values and the number of antibodies in which each mutation appears within the top 0.05% (third column). To consider common patterns across antibodies of the same class, we further retained only mutations shared by at least more than 10% of total antibodies within the top 0.05%. The numbers of preselected mutations in the top 0.05% are 455, 463, 463, 124, and 269 for V3-glycan, CD4bs, V2-apex, MPER, and Interface/FP bnAbs, respectively. (PDF) [file pcbi.1014095.s004.pdf]

Antibody class	Mutation	Number of observations within top 0.05% (Total number of antibodies)	Averaged contribution to log IC50 (absolute) within top 0.05%	Note
V3-glycan	K305	16 (110)	-0.23 (0.23)	K305, together with N300, is a critical residue within the V3 loop involved in interactions with the V2 loop and influences sensitivity to V3-glycan antibodies [1].
V3-glycan	H330	32 (110)	-0.33 (0.33)	Mutation H330Y increase resistance to V3-glycan antibodies [2, 3], and is located on the epitope of the V3-glycan antibody [4].
V3-glycan	Y330	31 (110)	0.29 (0.29)	Mutation H330Y increase resistance to V3-glycan antibodies [2, 3], and is located on the epitope of the V3-glycan antibody [4].
V3-glycan	O332	38 (110)	-0.33 (0.33)	Increase sensitivity to V3-glycan bnAbs, and is located on the V3-glycan antibody's epitope [3, 4]. Loss of O332 glycan enhances resistance to V3-glycan antibodies [5].
V3-glycan	S334	39 (110)	-0.31 (0.31)	Increase sensitivity to V3-glycan bnAbs [3]. Loss of S334 leads to an escape from V3-glycan bnAbs [6]
V3-glycan	O334	30 (110)	0.28 (0.28)	Increase resistance to V3-glycan bnAbs [2, 3].
V3-glycan	T336	14 (110)	0.26 (0.26)	Increase resistance to V3-glycan bnAbs [2, 3].
CD4BS	K178	20 (112)	-0.17 (0.17)	
CD4BS	D279	15 (112)	0.17 (0.21)	N279D, located on the CD4bs antibody epitope [4], moderately enhances sensitivity to CD4bs bnAbs [7], though its effects varies depends on the specific bnAbs [3].
CD4BS	N279	13 (112)	-0.18 (0.22)	N279K, located on the CD4bs antibody epitope [4], increases resistance to CD4bs, and contribute to escape 2BNC117, a CD4BS bnAb [8].
CD4BS	S364	15 (112)	-0.17 (0.17)	Located on the CD4bs antibody epitope [4], and increase sensitivity to CD4bs bnAbs [3].
CD4BS	E429	15 (112)	-0.18 (0.18)	Located on the CD4bs antibody epitope [4], sensitivity to CD4bs bnAbs can vary, either increasing or decreasing based on the specific bnAbs [3].
V2-Apex	G23	13 (112)	0.23 (0.23)	
V2-Apex	A30	14 (112)	0.23 (0.23)	
V2-Apex	O130	12 (112)	0.26 (0.26)	Located on the V2-apex antibody epitope [4], and sensitivity to V2-apex bnAbs can vary based on specific antibodies [9].
V2-Apex	Δ142-144	*17.3 (112)	-0.32 (0.32)	
V2-Apex	Q170	19 (112)	0.22 (0.24)	Located on the V2-apex antibody epitope [4], sensitivity to V2-apex bnAbs can vary based on specific antibodies [10, 4].
V2-Apex	Y173	17 (112)	0.24 (0.24)	Located on the V2-apex antibody epitope [4], appears to favorably orient the O156, which increase the sensitivity to VRC38.01, a V2-apex bnAb [9]. However, sensitivity to V2-apex bnAbs can vary, depending on bnAbs [3].
V2-Apex	K340	14 (112)	0.26 (0.26)	Enhances sensitivity to V2-apex bnAbs [3].
V2-Apex	O463	15 (112)	0.25 (0.25)	Enhances resistance to CD4BS antibodies [11].
V2-Apex	I515	14 (112)	0.28 (0.28)	
V2-Apex	L515	13 (112)	-0.22 (0.22)	
V2-Apex	N677	16 (112)	0.25 (0.25)	Enhances sensitivity to V2-apex bnAbs [3].
MPER	Q170	6 (30)	-0.13 (0.13)	
MPER	Y330	4 (30)	0.12 (0.12)	
MPER	S334	4 (30)	-0.14 (0.14)	
MPER	T676	5 (30)	-0.15 (0.15)	Enhances sensitivity to MPER bnAbs [3], and is necessary to recognize by 4E10, a specific MPER bnAb [12].
MPER	S676	4 (30)	0.15 (0.15)	Enhances resistance to MPER bnAbs [3], and loss of T676 leads to reduction in binding to 4E10 (ref. [12]) .
MPER	N677	4 (30)	0.15 (0.15)	Loss of N677 can contribute to enhanced resistance [13]
MPER	I829	8 (30)	0.13 (0.13)	
MPER	L832	4 (30)	0.12 (0.12)	
IF/FP	D279	11 (65)	-0.21 (0.21)	
IF/FP	N279	9 (65)	-0.20 (0.20)	
IF/FP	N300	8 (65)	-0.19 (0.19)	
IF/FP	I515	10 (65)	-0.27 (0.27)	Located on the fusion peptide antibody epitope [4], and enhances sensitivity to IF/FP bnAbs [3], especially VRC34.01 bnAb [14, 15]. Some mutations such as Lys of I515 leads to enhanced resistance to FP bnAbs [4, 16].
IF/FP	L515	8 (65)	0.26 (0.26)	Located on the fusion peptide antibody epitope [4], and enhances resistance to FP bnAbs [16].
IF/FP	L815	7 (65)	0.19 (0.19)	

S3 Table Mutations that are shared across more than 10% of analyzed antibodies and ranked top 0.05%. This table summarizes key mutations identified based on their contributions to the predicted log IC50 values. Mutations are selected from the top 0.05% according to the absolute values of their weight parameters, which quantify their contributions to the predicted log IC50, across all antibodies within the same antibody class. For these top-ranked mutations, the table reports the average contribution values and the number of antibodies in which each mutation appears within the top 0.05% (third column). To consider common patterns across antibodies of the same class, we further retained only mutations shared by at least more than 10% of total antibodies within the top 0.05%. The numbers of preselected mutations in the top 0.05% are 455, 463, 463, 124, and 269 for V3-glycan, CD4bs, V2-apex, MPER, and Interface/FP bnAbs, respectively.

References

[1] Christina Guzzo et al. “Structural constraints at the trimer apex stabilize the HIV-1 envelope in a closed, antibody-protected conformation”. In: *Mbio* 9.6 (2018), pp. 10–1128.

[2] Panagiota Zacharopoulou et al. “Prevalence of resistance-associated viral variants to the HIV-specific broadly neutralising antibody 10-1074 in a UK bNAb-naıve population”. In: *Frontiers in Immunology* 15 (2024), p. 1352123.

[3] Christine A Bricault et al. “HIV-1 neutralizing antibody signatures and application to epitope-targeted vaccine design”. In: *Cell host & microbe* 25.1 (2019), pp. 59–72.

[4] Gwo-Yu Chuang et al. “Structural survey of broadly neutralizing antibodies targeting the HIV-1 Env trimer delineates epitope categories and characteristics of recognition”. In: *Structure* 27.1 (2019), pp. 196–206.

[5] DR Martinez et al. *Maternal broadly neutralizing antibodies can select for neutralization-resistant, infant-transmitted/founder HIV variants. mBio 11: e00176-20.* 2020.

[6] Caelan E Radford and Jesse D Bloom. “Comprehensive maps of escape mutations from antibodies 10-1074 and 3BNC117 for Envs from two divergent HIV strains”. In: *Journal of Virology* 99.5 (2025), e00195–25.

[7] Paula Cohen et al. “Resistance mutations that distinguish HIV-1 envelopes with discordant VRC01 phenotypes from multi-lineage infections in the HVTN703/HPTN081 trial: implications for cross-resistance”. In: *Journal of Virology* 99.2 (2025), e01730–24.

[8] Lutz Gieselmann et al. “Profiling of HIV-1 elite neutralizer cohort reveals a CD4bs bnAb for HIV-1 prevention and therapy”. In: *Nature Immunology* (2025), pp. 1–14.

[9] Evan M Cale et al. “Virus-like particles identify an HIV V1V2 apex-binding neutralizing antibody that lacks a protruding loop”. In: *Immunity* 46.5 (2017), pp. 777–791.

[10] Rebecca T van Dorsten et al. “Neutralization breadth and potency of single-chain variable fragments derived from broadly neutralizing antibodies targeting multiple epitopes on the HIV-1 envelope”. In: *Journal of Virology* 94.2 (2020), pp. 10–1128.

[11] Wenbo Wang et al. “N463 glycosylation site on V5 loop of a mutant gp120 regulates the sensitivity of HIV-1 to neutralizing monoclonal antibodies VRC01/03”. In: *JAIDS Journal of Acquired Immune Deficiency Syndromes* 69.3 (2015), pp. 270–277.

[12] Florence M Brunel et al. “Structure-function analysis of the epitope for 4E10, a broadly neutralizing human immunodeficiency virus type 1 antibody”. In: *Journal of virology* 80.4 (2006), pp. 1680–1687.

[13] Saikat Banerjee et al. “Evaluation of a novel multi-immunogen vaccine strategy for targeting 4E10/10E8 neutralizing epitopes on HIV-1 gp41 membrane proximal external region”. In: *Virology* 505 (2017), pp. 113–126.

[14] Meng Yuan et al. “Conformational plasticity in the HIV-1 fusion peptide facilitates recognition by broadly neutralizing antibodies”. In: *Cell Host & Microbe* 25.6 (2019), pp. 873–883.

[15] Rui Kong et al. “Fusion peptide of HIV-1 as a site of vulnerability to neutralizing antibody”. In: *Science* 352.6287 (2016), pp. 828–833.

[16] Adam S Dingens et al. “Complete functional mapping of infection-and vaccine-elicited antibodies against the fusion peptide of HIV”. In: *PLoS pathogens* 14.7 (2018), e1007159.
