# Supplementary material for: Predicting viral sensitivity to antibodies using genetic sequences and antibody similarities
Source: PLoS Comput Biol. 2026 Mar 23;22(3):e1014095. doi: 10.1371/journal.pcbi.1014095 (PMC13020759; doi:10.1371/journal.pcbi.1014095)
Supplement: S4 Table — The mean cross-validation-based Pearson’s R (CV-R) values for each antibody type are listed in the table, with individual CV-R values shown in Fig 6 of the main text. The mean values were calculated only for bnAbs with sufficient data to allow SLAPNAP to run. For V1V2, V3, CD4bs, and MPER bnAbs, the mean CV-R values of GNL are often more than 10% higher than those of other methods. The overall mean CV-R, averaged across antibody types, indicates that GNL achieves the highest accuracy, with approximately a 10% improvement. (PDF) [file pcbi.1014095.s005.pdf]

Type of bnAbs	Einav et al. [1]	SLAPNAP [2]	GNL (our method)
V1V2	0.33	0.53	**0.63**
V3	0.51	0.55	**0.63**
CD4bs	0.52	0.46	**0.58**
Fusion peptide	0.32	**0.55**	0.52
Subunit interface	0.21	**0.43**	0.34
MPER	0.65	0.59	**0.75**
Total	0.46	0.51	**0.60**

S4 Table Mean Pearson's R values based on cross-validation The mean cross-validation-based Pearson's R (CV-R) values for each antibody type are listed in the table, with individual CV-R values shown in **Fig. 6** of the main text. The mean values were calculated only for bnAbs with sufficient data to allow SLAPNAP to run. For V1V2, V3, CD4bs, and MPER bnAbs, the mean CV-R values of GNL are often more than 10% higher than those of other methods. The overall mean CV-R, averaged across antibody types, indicates that GNL achieves the highest accuracy, with approximately a 10% improvement.

References

[1] Tal Einav and Brian Cleary. "Extrapolating missing antibody-virus measurements across serological studies". In: *Cell Systems* 13.7 (2022), pp. 561–573.

[2] Brian D Williamson et al. "Super LeArner Prediction of NAb Panels (SLAPNAP): a containerized tool for predicting combination monoclonal broadly neutralizing antibody sensitivity". In: *Bioinformatics* 37.22 (2021), pp. 4187–4192.
