# Supplementary material for: Predicting viral sensitivity to antibodies using genetic sequences and antibody similarities
Source: PLoS Comput Biol. 2026 Mar 23;22(3):e1014095. doi: 10.1371/journal.pcbi.1014095 (PMC13020759; doi:10.1371/journal.pcbi.1014095)
Supplement: S5 Table — Antibodies marked with an asterisk (*) are classified differently in the literature. While earlier studies did not classify them as bnAbs [27,51], more recent research considers them to be bnAbs [52,53]. (PDF) [file pcbi.1014095.s006.pdf]

ID in this study	Antibody name	Condition
1	CH103	mature bnAb
2	CH104	mature bnAb
3	CH105	mature bnAb
4	CH106	mature bnAb
5	CH103.IA1	early/intermediate lineage
6	CH103.IA2	early/intermediate lineage
7	CH103.IA3	early/intermediate lineage
8	CH103.IA4	early/intermediate lineage
9	CH103.IA5	early/intermediate lineage
10	CH103.IA6	early/intermediate lineage
11	CH103.IA7	early/intermediate lineage
12	CH103.IA8	early/intermediate lineage
13	CH235	mature bnAb*
14	CH236	mature bnAb*
15	CH239	mature bnAb*
16	CH240	mature bnAb*
17	CH241	mature bnAb*
18	CH235.IA1	early/intermediate lineage
19	CH235.IA2	early/intermediate lineage
20	CH235.IA3	early/intermediate lineage
21	CH235.IA4	early/intermediate lineage

S5 Table Antibodies from intrahost HIV-1 evolution data[1]. * Classification of these antibodies varies across the literature; they were not classified as bnAbs in earlier work[1, 2]; however, other studies, such as recent studies, consider them to be bnAbs [3, 4].

References

[1] Feng Gao et al. "Cooperation of B cell lineages in induction of HIV-1-broadly neutralizing antibodies". In: *Cell* 158.3 (2014), pp. 481–491.

[2] Mattia Bonsignori et al. "Maturation pathway from germline to broad HIV-1 neutralizer of a CD4-mimic antibody". In: *Cell* 165.2 (2016), pp. 449–463.

[3] Kevin O Saunders et al. "Targeted selection of HIV-specific antibody mutations by engineering B cell maturation". In: *Science* 366.6470 (2019), eaay7199.

[4] Christoph Kreer et al. "Probabilities of developing HIV-1 bNAb sequence features in uninfected and chronically infected individuals". In: *Nature communications* 14.1 (2023), p. 7137.
