# Supplementary figures and images for: Predicting viral sensitivity to antibodies using genetic sequences and antibody similarities
Source: PLoS Comput Biol. 2026 Mar 23;22(3):e1014095. doi: 10.1371/journal.pcbi.1014095 (PMC13020759; doi:10.1371/journal.pcbi.1014095)

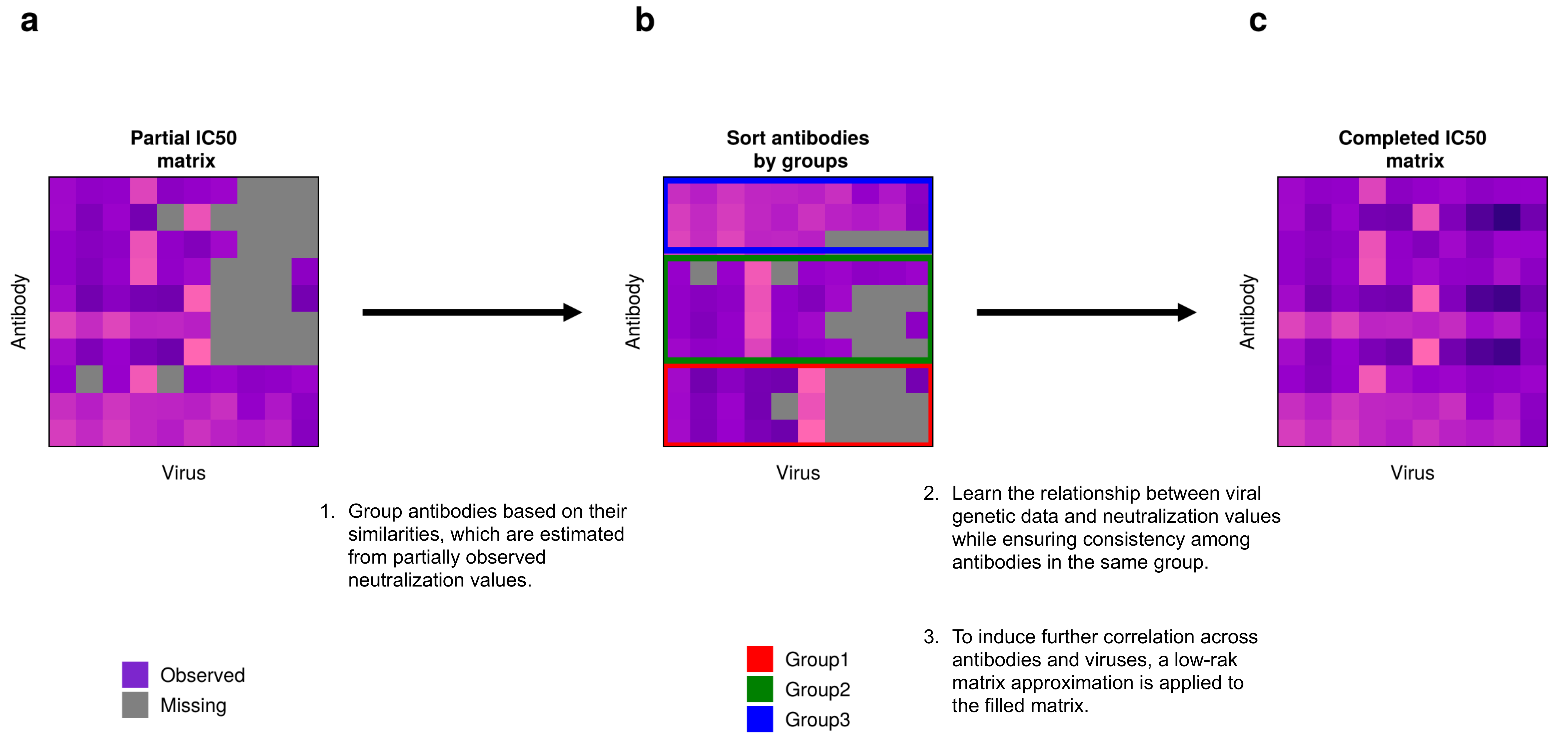

Supplement: S1 Fig — (a) The partially observed neutralization matrix (columns: viruses, rows: antibodies) is used as input to impute the missing elements (gray scale) of the neutralization matrix. (b) Schematic representation of grouping antibodies based on the similarity of their neutralization values. Within the same group, neutralization activities are more similar than those between different groups. (c) Temporarily complete missing values using models that learn the relationship between viral genetic data and neutralization values. We employ the novel grouped learning method, training the model to simultaneously learn the relationships between neutralization values and viral genetic sequences for a group of antibodies that share similar neutralization profiles (S1 Text). Here, to effectively reduce the number of model parameters and avoid the common over-parametrization issue, we applied a standard dimensional reduction technique to viral sequences and trained the model using the projected sequences. (d) Obtain the complete neutralization matrix, derived from the low-rank matrix approximation of the temporarily completed matrix from step c. The optimal matrix rank was determined based on the distribution of eigenvalues (S1 Text). (TIFF) [file pcbi.1014095.s007.tiff]

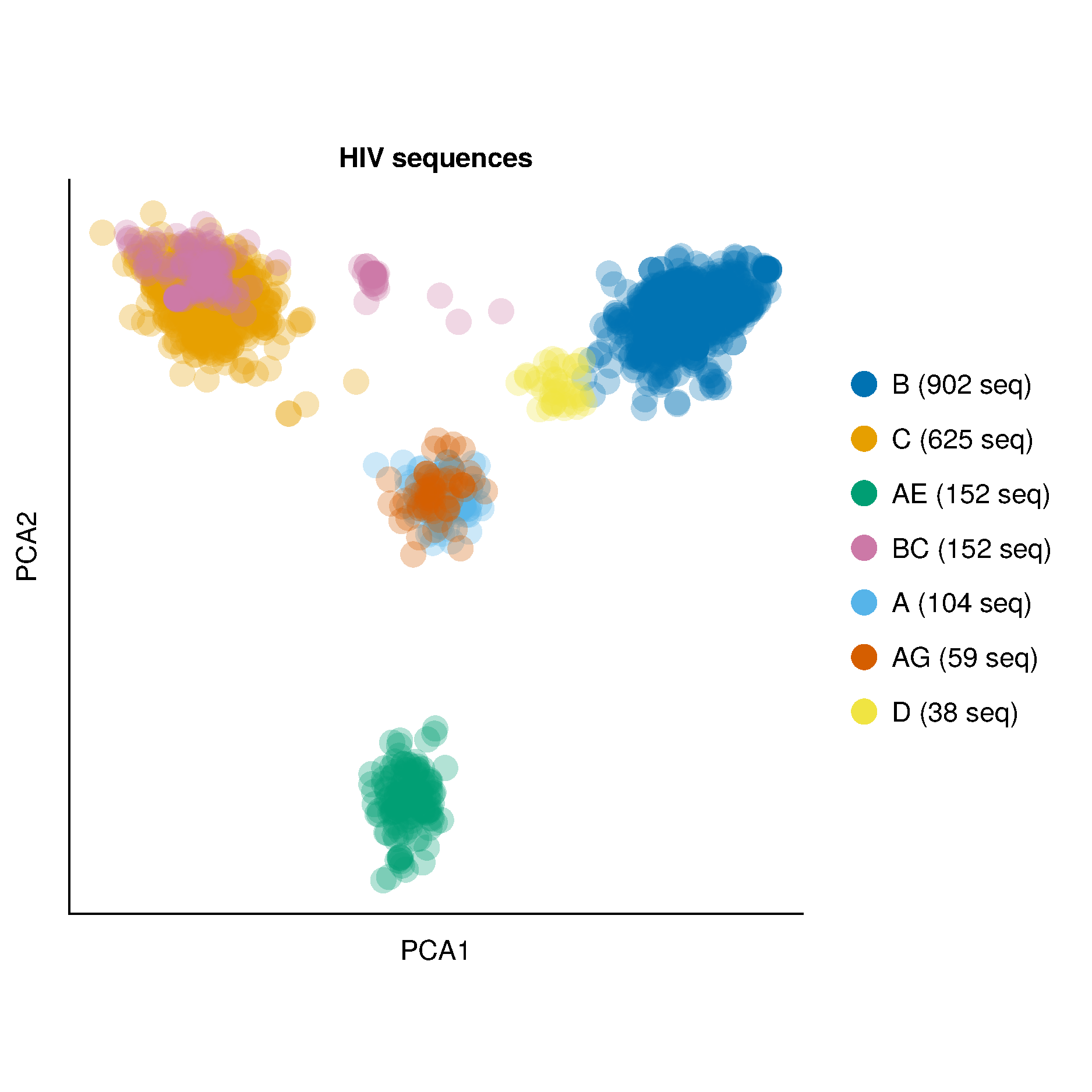

Supplement: S2 Fig — Each point in the cloud represents a viral sequence projected onto the principal modes of the covariance matrix derived from the one-hot encoded sequences. These principal modes yield projected sequences that are also used to learn the neutralization values. In this visualization, we present only subtypes that include more than 30 viral sequences that are used in our training data. Subtypes such as B, C, A, and AE are mapped to distinct regions in the space. (TIF) [file pcbi.1014095.s008.tif]

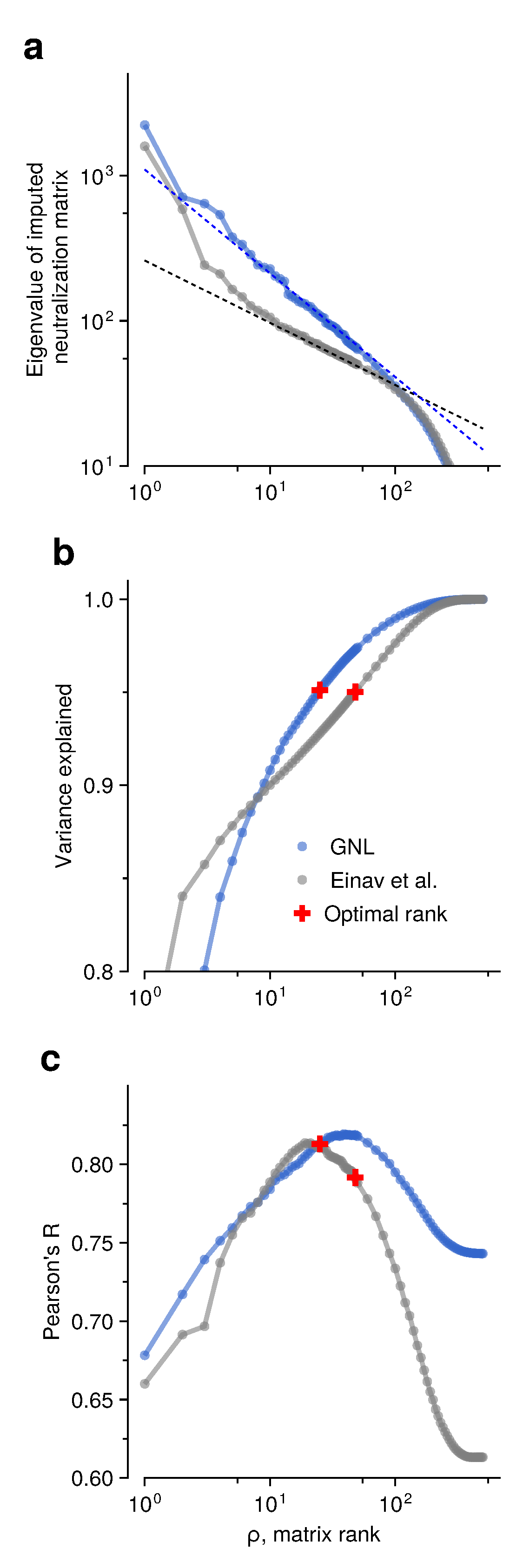

Supplement: S3 Fig — As a typical example, we set the fraction of observed data as 80%, and the validation values were withheld uniformly at random from the CATNAP dataset. (a) Eigenvalues for the filled neutralization matrices in the GNL and Einav et al. methods. The intermediate ranks of eigenvalues roughly follow a power law. Dashed lines show the power law fit with exponents of −3/7 and −5/7, respectively. (b) Profiles of the variance explained for GNL and Einav et al. methods. The “optimal” matrix rank values, defined as the minimum rank at which the explained variance exceeds 95%, are 20 and 45 for the GNL and Einav et al. methods, respectively. (c) Pearson’s R between true and predicted neutralization values shown as a function of rank. Including additional eigenmodes ultimately reduces predictive power. The rate of decrease in Pearson’s R using the GNL method is more gradual than that of Einav et al., and it maintains higher values. (TIF) [file pcbi.1014095.s009.tif]

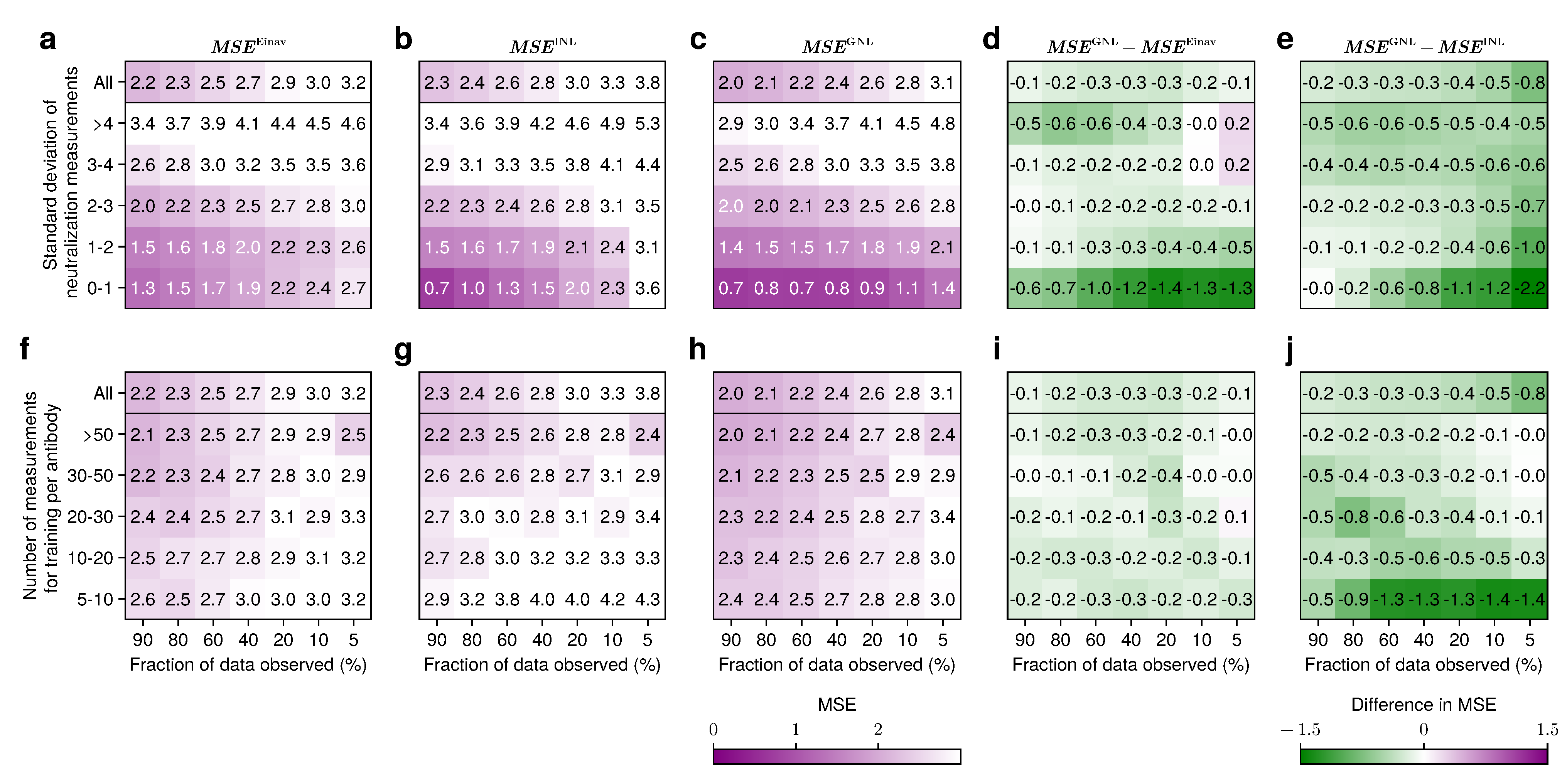

Supplement: S4 Fig — (d, e) Differences in MSE values between GNL and the other methods. GNL outperforms INL for antibodies with higher variability in neutralization and for lower fractions of observed data (e), suggesting that antibody grouping mechanisms aid in learning from variable cases. (f-h) MSE values across different numbers of training measurements and (i, j) their corresponding MSE differences. The grouping method benefits antibodies with fewer observed measurements, suggesting the advantage of grouping for sparsely observed antibody cases (j). (TIF) [file pcbi.1014095.s010.tif]

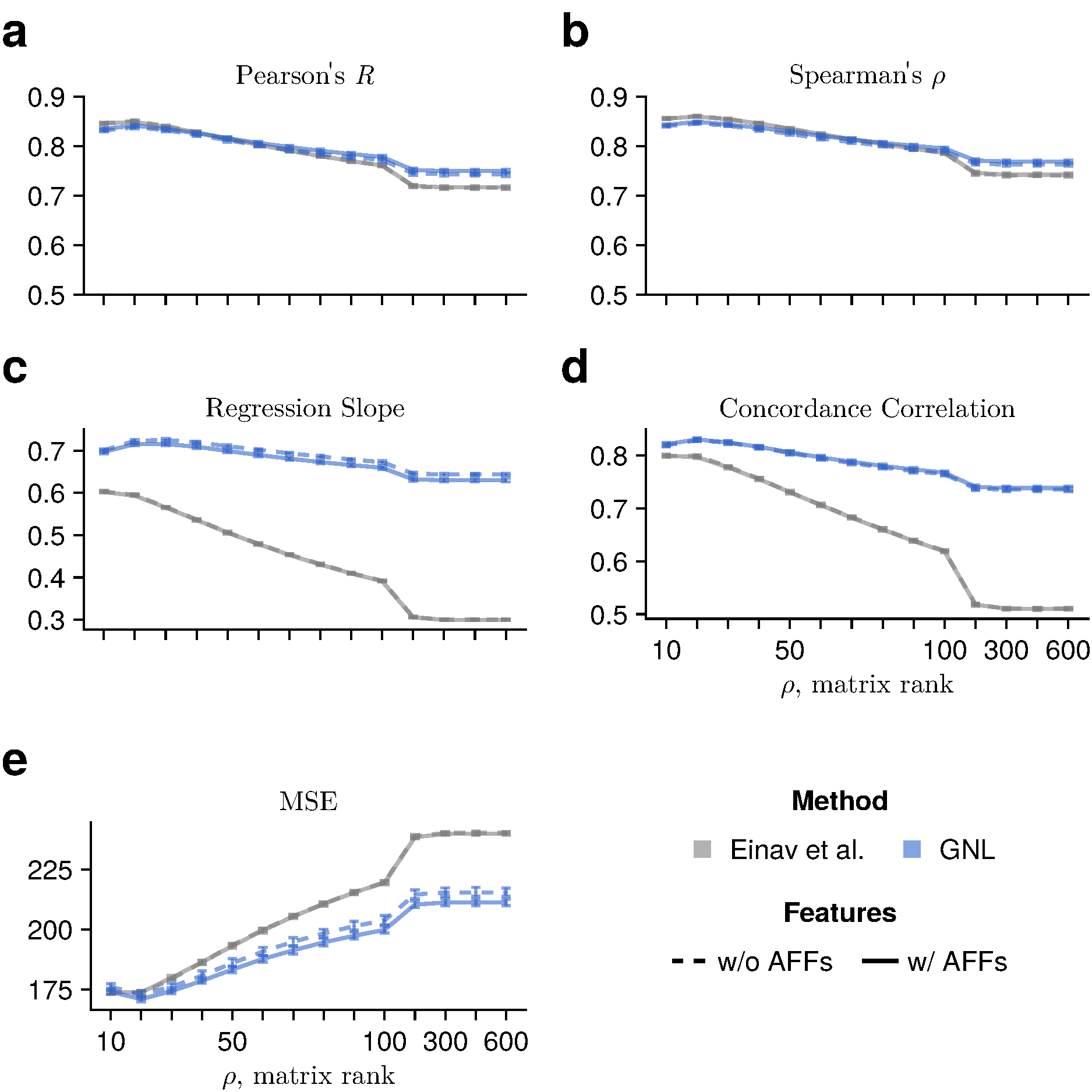

Supplement: S5 Fig — The same experimental conditions as in Fig 3 of the main text are used, with IC80 considered as an alternative viral sensitivity measure. Multiple accuracy metrics are used: (a) Pearson’s R, (b) Spearman’s ρ, (c) regression slope, (d) concordance correlation coefficients, and (e) MSE. Solid and dashed lines represent results from models trained with both sequence alignments and alignment-free features (AFFs), and with sequence alignments alone, respectively. (TIF) [file pcbi.1014095.s011.tif]

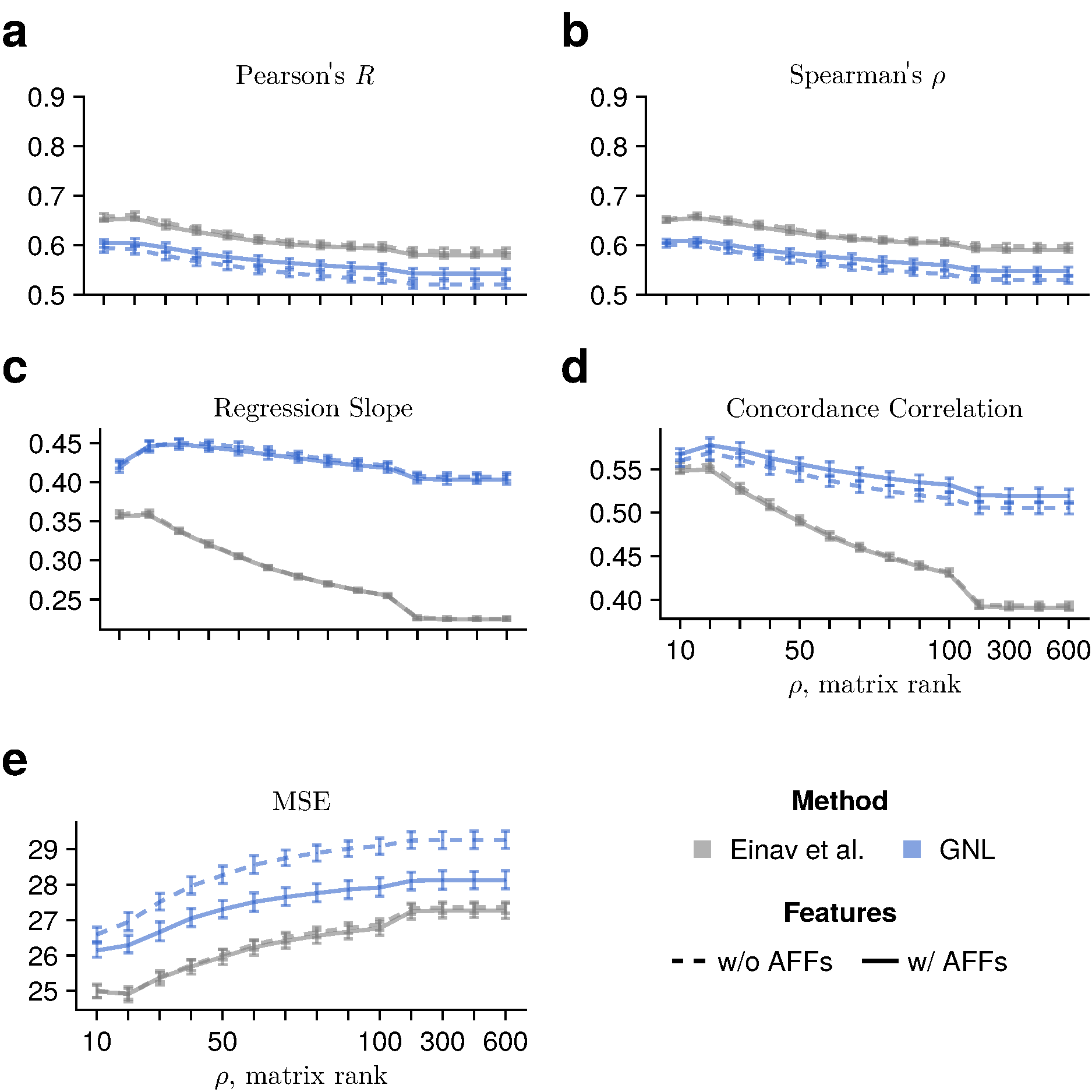

Supplement: S6 Fig — (TIF) [file pcbi.1014095.s012.tif]

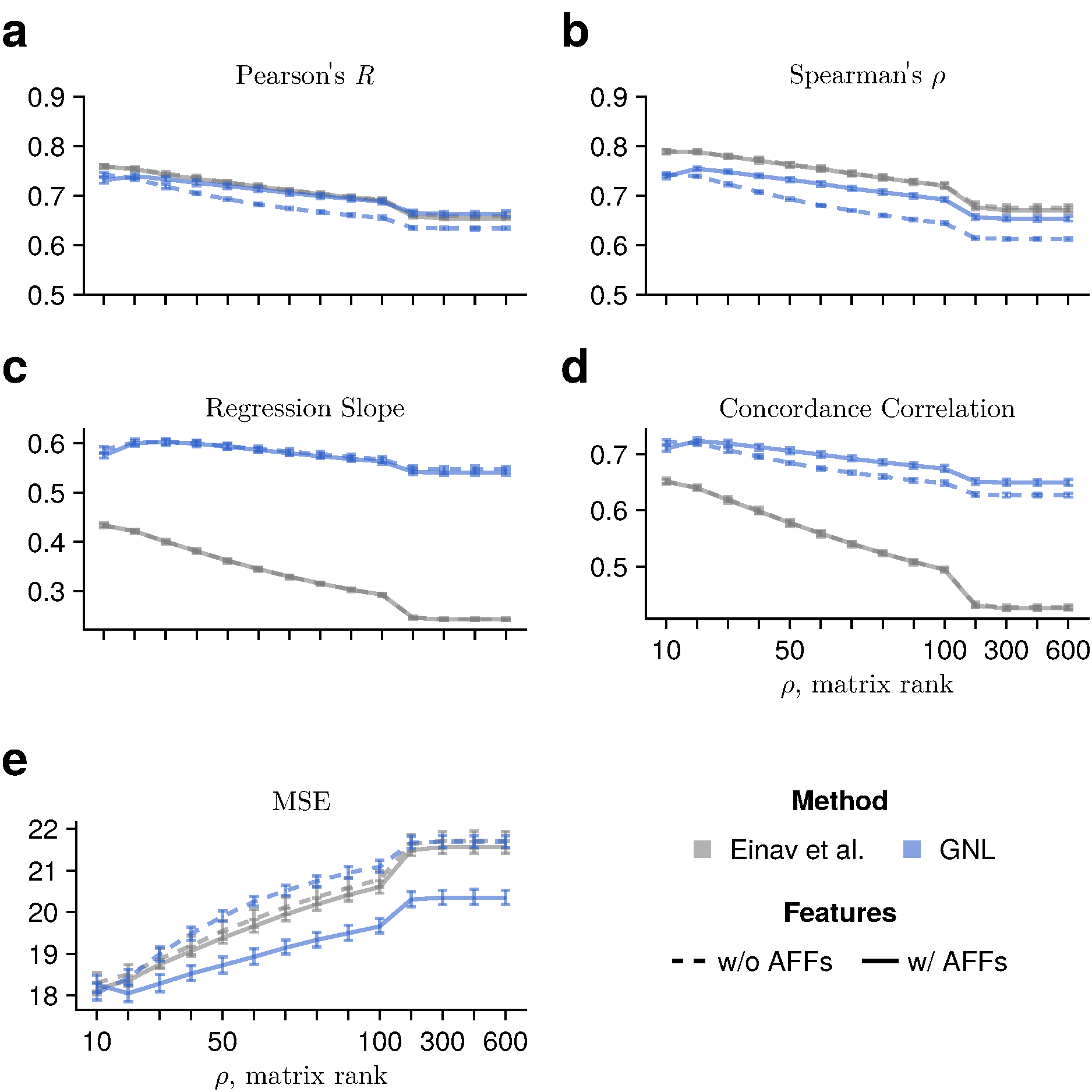

Supplement: S7 Fig — (TIF) [file pcbi.1014095.s013.tif]

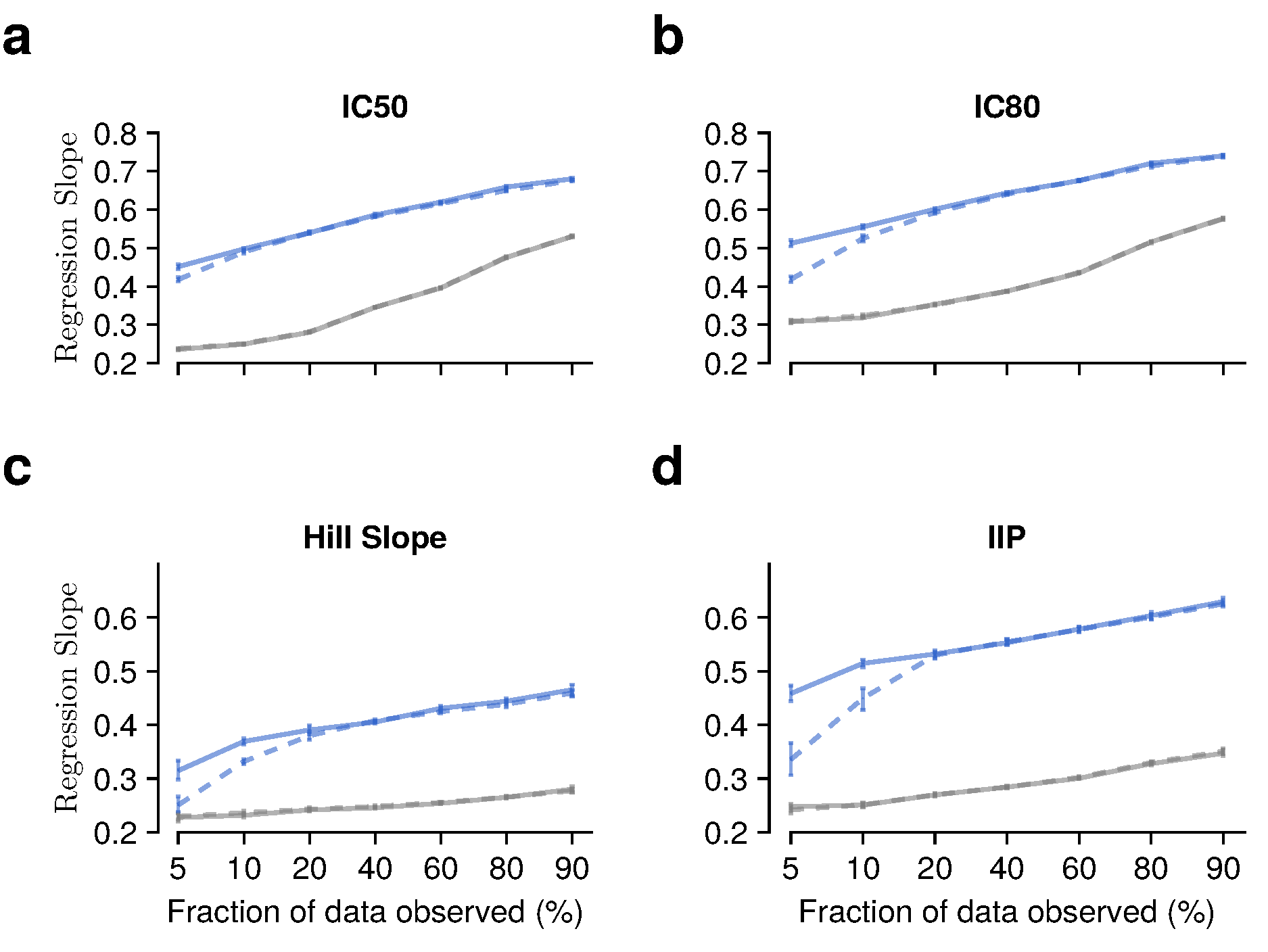

Supplement: S8 Fig — (TIF) [file pcbi.1014095.s014.tif]

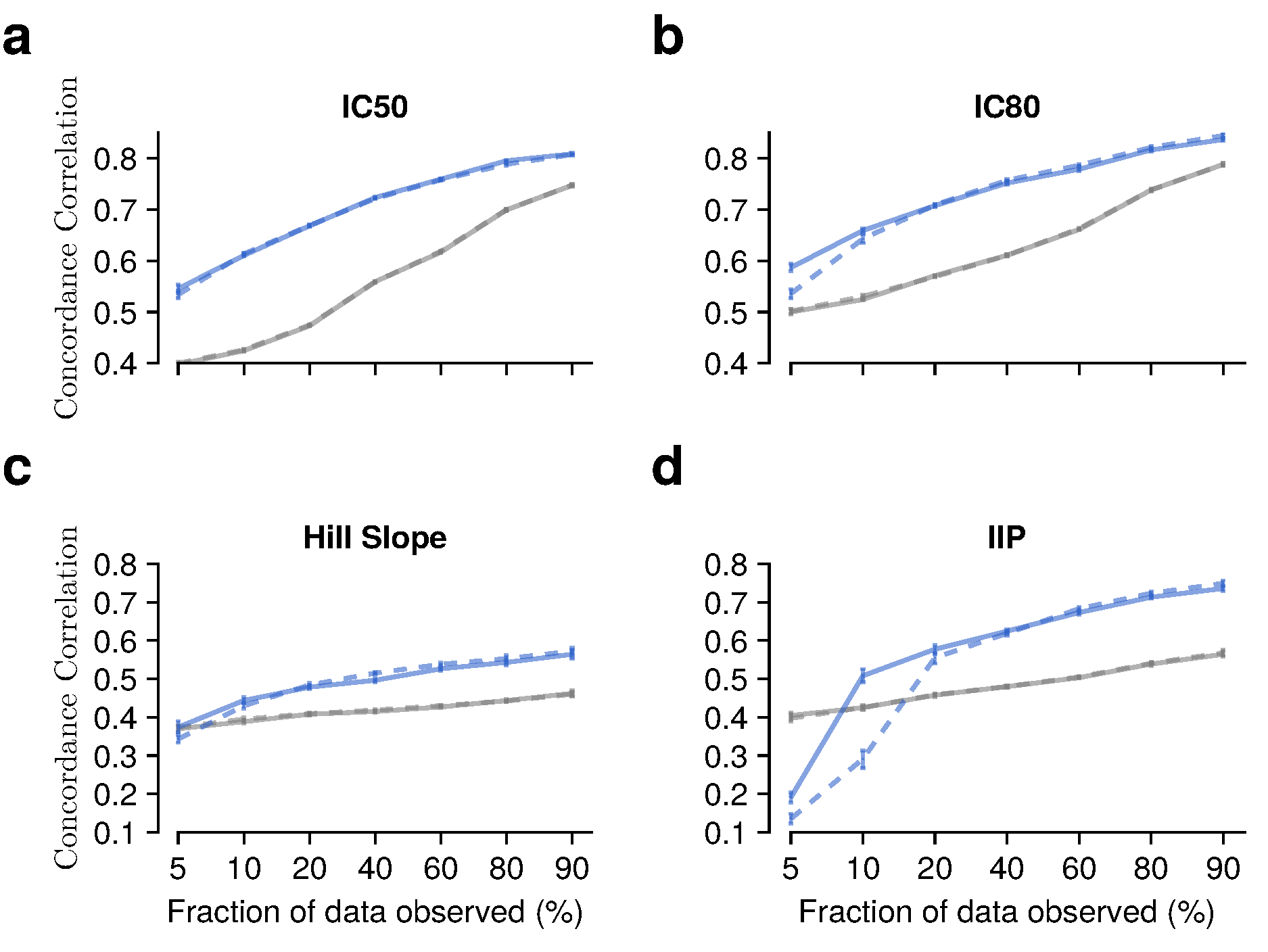

Supplement: S9 Fig — Multiple viral sensitivity measures are considered: (a) IC50, (b) IC80, (c) Hill slope, (d) IIP, which was evaluated at a fixed antibody concentration of c = 1000 μg/mL. (TIF) [file pcbi.1014095.s015.tif]

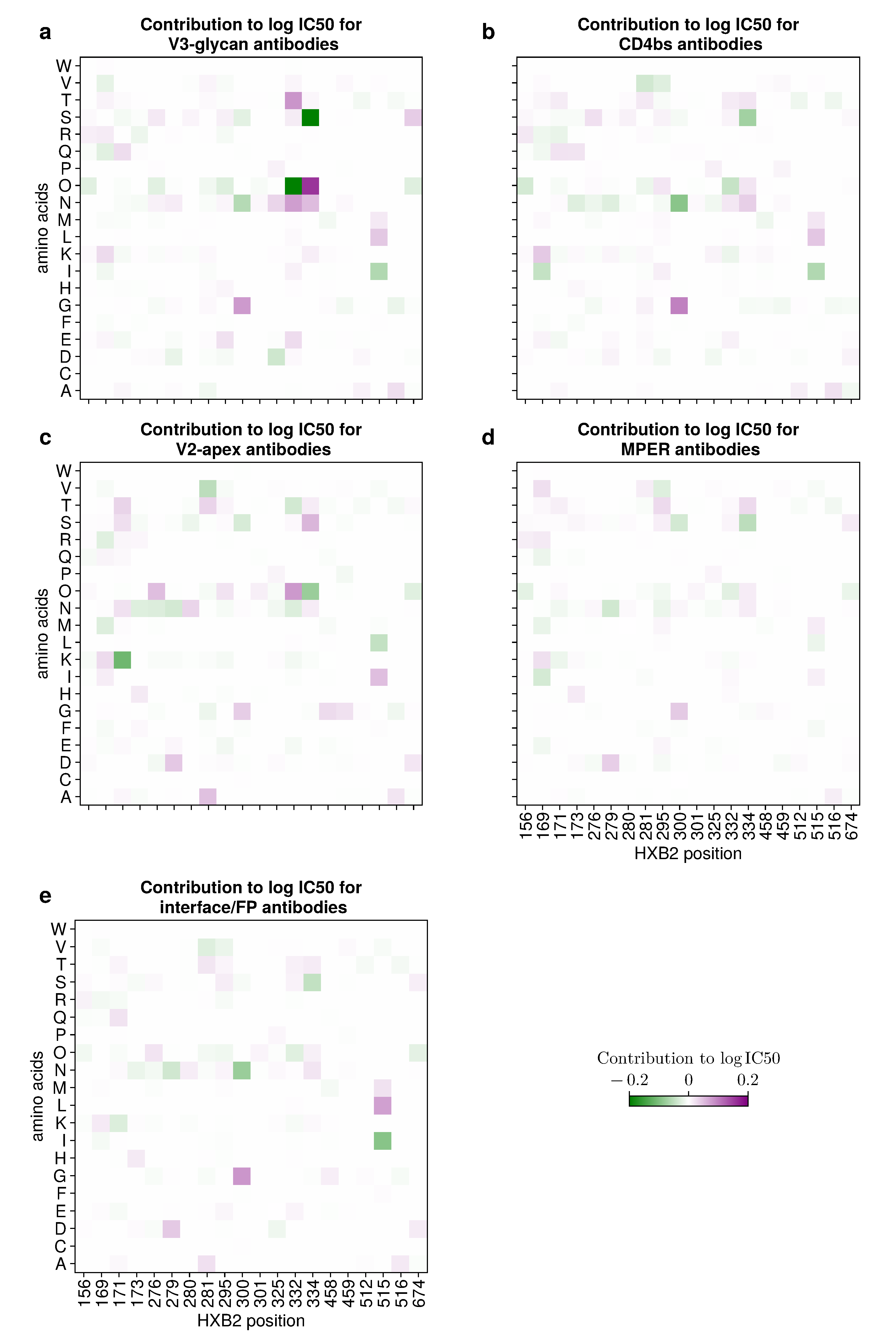

Supplement: S10 Fig — The experimental setup is the same as in Fig 5 of the main text. Contributions to log(IC50) are obtained by averaging across multiple antibodies within the same antibody class. The numbers of antibodies included in this analysis are 110, 112, 112, 30, and 65 for (a) V3-glycan, (b) CD4bs, (c) V2-apex, (d) MPER, and (e) Interface/FP antibodies, respectively. Mutation sites are selected based on experimental studies [28,31–36]. (TIF) [file pcbi.1014095.s016.tif]

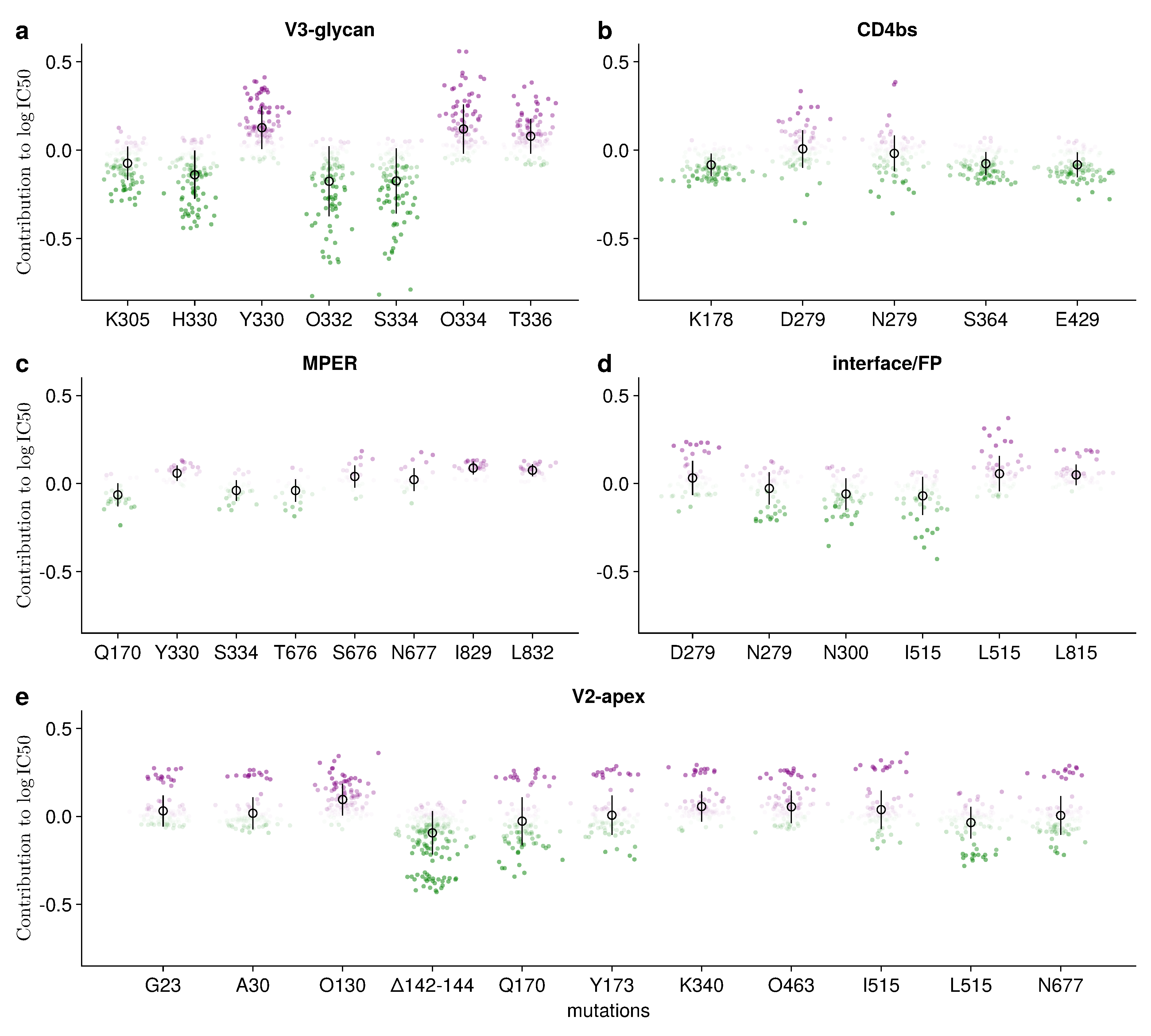

Supplement: S11 Fig — Mutations are selected by ranking the absolute values of their weight parameters, which represent contributions to the predicted log IC50 values, and retaining those within the top 0.05%. These mutations are then filtered to include only those present in more than 10% of antibodies within the same antibody class. (However, the points shown represent weight values across all antibodies, regardless of whether they fall within or outside the top 0.05% selection.) Detailed statistics and the effects of these mutations on antibody neutralization are summarized in S3 Table. (TIF) [file pcbi.1014095.s017.tif]

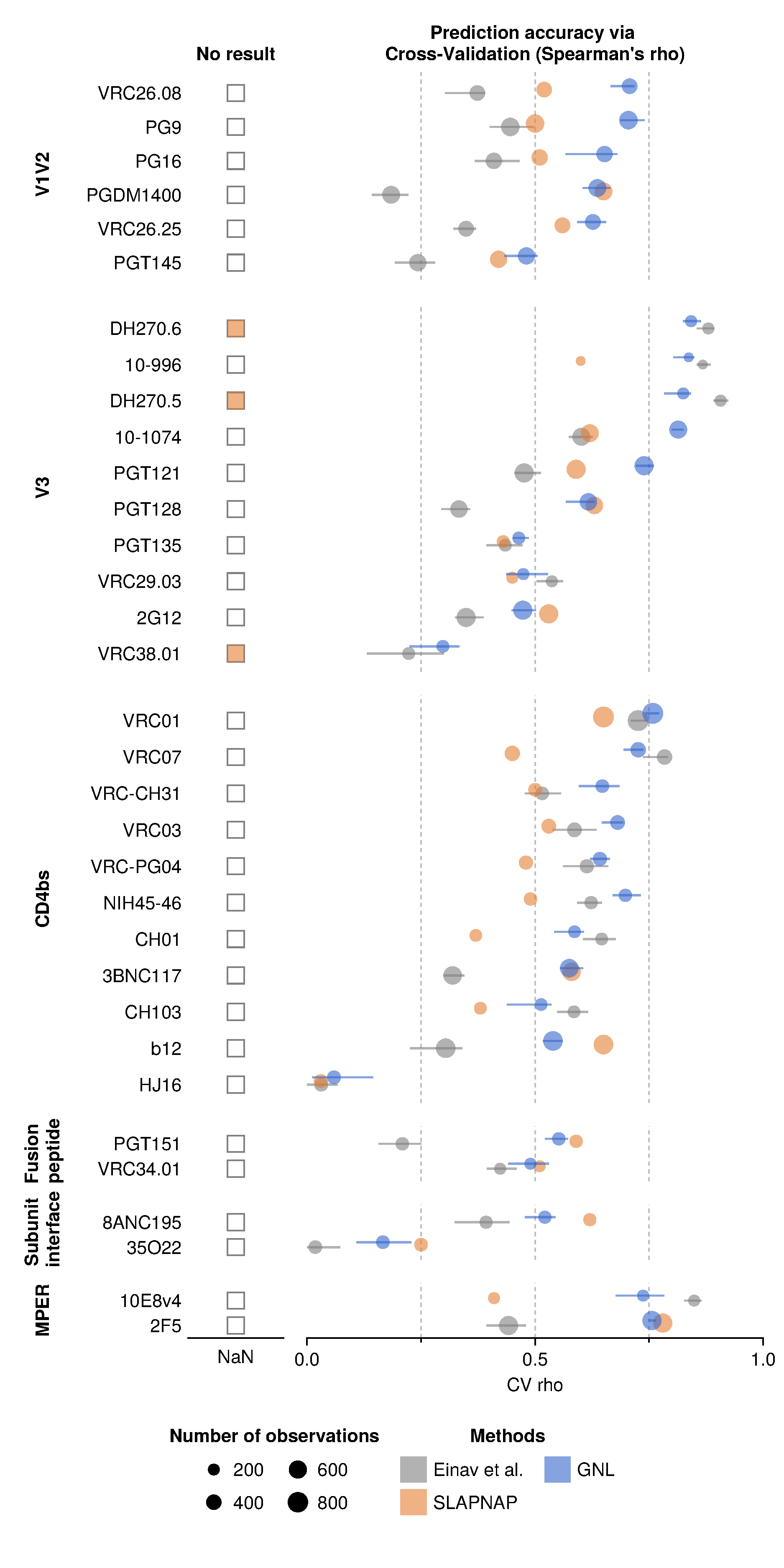

Supplement: S12 Fig — The same experimental setup as in Fig 6 of the main text is used, with results shown for cross-validation–based Spearman’s correlation. (TIF) [file pcbi.1014095.s018.tif]

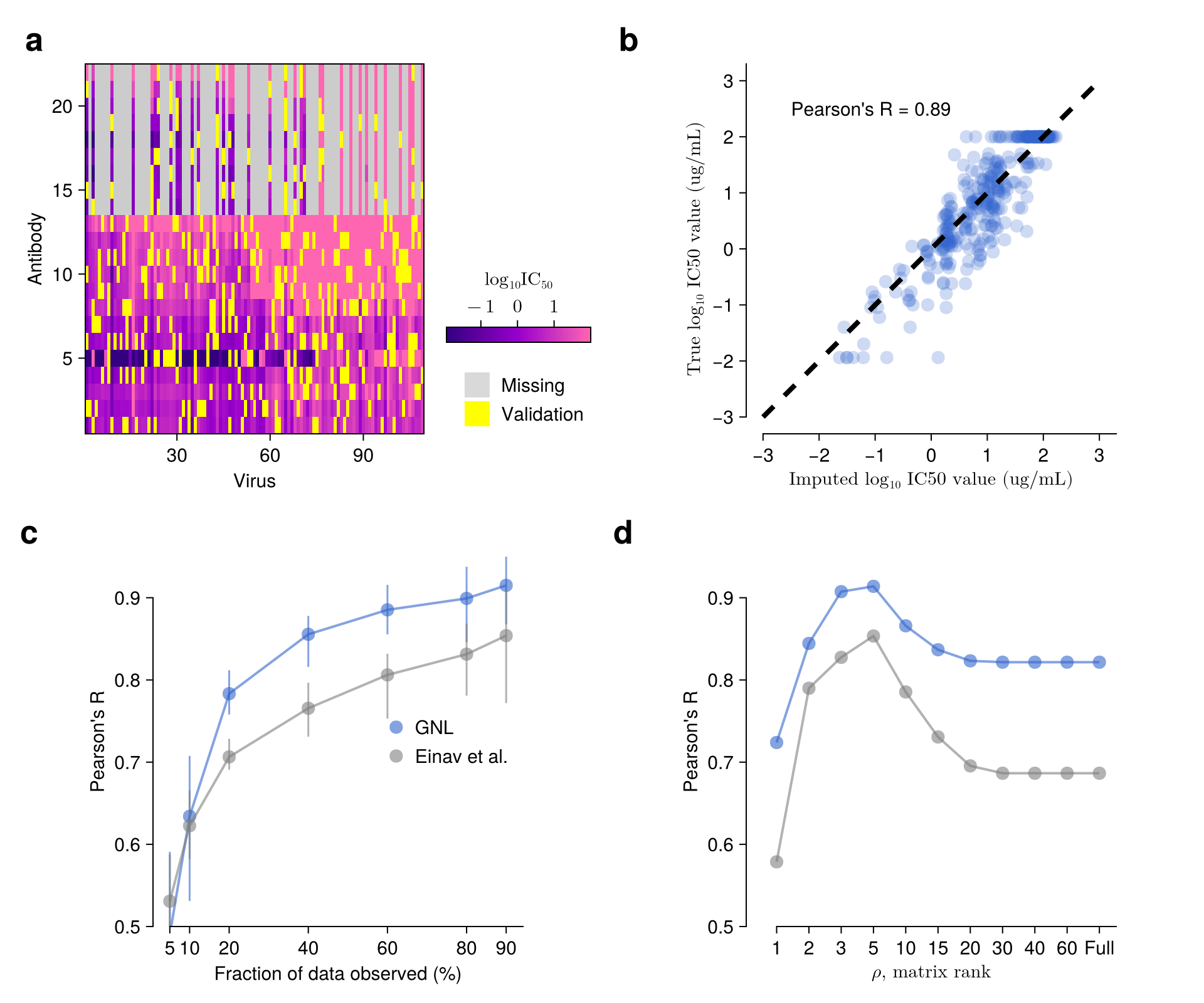

Supplement: S13 Fig — (a) Neutralization values are represented with purple, gray, and yellow colors, corresponding to observed, missing, and withheld values for validation, respectively. The withheld elements are chosen uniformly at random across antibodies and viruses. In this analysis, 80% of the total available data is observed. (b) A comparison of the true withheld neutralization values and the imputed values is shown on the x- and y-axes. Pearson’s R, Spearman’s ρ, MSE, and p− values are, 0.89, 0.82, 9.42, and 10−90.9, respectively. (c) Accuracy Dependency on the Fraction of Observed Data. The overall accuracy of the GNL method is higher than that of the Einav et al. method (provided by Einav et al. [20]) as the fraction of observed data increases. (d) Dependency of accuracy on matrix rank ρ. The R values of the GNL method are consistently higher than those of the Einav et al. method across most rank values. (TIFF) [file pcbi.1014095.s019.tiff]
