# Supplementary material for: Predicting viral sensitivity to antibodies using genetic sequences and antibody similarities
Source: PLoS Comput Biol. 2026 Mar 23;22(3):e1014095. doi: 10.1371/journal.pcbi.1014095 (PMC13020759; doi:10.1371/journal.pcbi.1014095)
Supplement: Attachment — Submitted filename: 2026-02-10-response.pdf [file pcbi.1014095.s021.pdf]

RESPONSE TO REVIEWERS' COMMENTS

We thank the reviewers for a careful review and constructive comments, which have helped to strengthen our manuscript. Below, we respond to the reviewers' comments and describe our modifications to the manuscript. The reviewers' comments are reproduced in **bold** and our modifications or additions are written in blue.

REVIEWER 1

Shimagaki et al. have developed a computational approach to predict log(IC50) for anti-HIV antibodies based on the semi-large existing data sets for virus/mab pairs. This is an admirable goal and could be valuable to a range of prevention/therapy/and cure studies for HIV and beyond. The work is well validated against existing approaches and appears substantially better.

A few bigger picture comments:

A main stated motivation for this work is that neutralization assays are hard or not available for many bnAb virus combinations. But it might be worth it to mention the type of sequencing that is required to generate data for this method and how much easier/cheaper that is to give a reader a concrete demonstration of how useful this would be and how this would fit into a clinical study (for instance, in participant inclusion criterion after a quick sequencing step before an inteventional study?)

Thank you, this is an important practical point. We have now expanded the Introduction to elaborate:

> Because GNL requires only a reference neutralization database (e.g., CATNAP) and viral genetic sequence data (here, HIV-1 Env), it can provide rapid sensitivity estimates without performing new neutralization assays for each virus-antibody pair. In many study settings, Env sequencing can be generated routinely from participant samples and used to screen circulating or within-host variants. Computational approaches like GNL could therefore enable sequence-based sensitivity estimates to support tasks such as cohort stratification, prioritization of candidate bnAbs, or screening potential clinical trial participants when experimental neutralization testing is impractical.

It should be acknowledged that even if prediction is possible, unknown unknown HIV variants are practically problematic. Whether in circulation or within host such variants are potentially more relevant than more abundant variants -- historic work (Lorenzi et al) have shown abundant clonotypes are paradoxically less likely to drive relevant dynamics

Yes, we agree that prediction for previously unseen or rare variants could be challenging, even though they may be biologically important. We also note that this limitation applies both to circulating viral diversity and to within-host variation, and is

not unique to our approach but common to data-driven prediction methods. We have now added a line in the Discussion to make this point:

> First, as with any sequence-based prediction approach, our method is limited by the variants observed in the training data; rare or unobserved variants, which can be clinically important drivers of escape, might not be explicitly captured.

It might be useful to include further notions of sensitivity from CATNAP (i.e. hill slope), since you have those data. This would also enhance the novelty beyond the Einav et al paper. One choice could be IIP at a relevant dose level, perhaps 1000μg/mL or something? You can find equation here (https://www.nature.com/articles/s41467-023-43384-y) and we found IIP correlating with viral load in HIV prevention studies with VRC01, also you might cite Laskey and Siliciano etc if you use that measure.

Following the reviewer's suggestion, we expanded our analysis to include additional neutralization-related quantities, including IC80 values, Hill slope, and instantaneous inhibitory potential (IIP) computed at a concentration of 1000 μg/mL.

Revised text and analyses: In the revised manuscript, we evaluate prediction performance for these quantities alongside IC50. We find that the qualitative trends observed for IC50 largely carry over to IC80, with low-rank approximation consistently improving prediction accuracy across data fractions. In contrast, Hill slope and IIP values are more challenging to predict: while GNL outperforms the Einav et al. method for IC50 and IC80, the two approaches achieve comparable performance for Hill slope and IIP when assessed using correlation-based metrics. For agreement-based metrics (regression slope and concordance correlation coefficient), GNL improved performance relative to the Einav et al. approach.

These results clarify both the strengths and limitations of sequence-based prediction for different notions of neutralization sensitivity and help delineate which metrics can be reliably inferred from existing data. Optimization specifically for other neutralization metrics may also improve performance. We have now added a new section to the paper, **Predicting viral sensitivity metrics beyond IC50**, which describes these analyses and an interpretation of the results, included below for completeness:

> Overall, we observed consistent trends in the relationship between observed fraction and accuracy (**Fig. 4**), the relationship between prediction accuracy and matrix rank, with reduced matrix rank generally improving prediction accuracy (**Supplementary Fig. 7**). Our method is robust to the choice of matrix rank, and Pearson's R exceeds 0.8 across a wide range of rank values when 80% of the data are observed. However, we note that prediction accuracy for Hill slope and IIP values is relatively insensitive to matrix rank (**Supplementary Figs. 7-9**), and that GNL's Pearson and Spearman correlation coefficients are comparable to or slightly lower than those obtained

using the method of Einav et al. In contrast, for the regression slope and concordance correlation coefficient, the GNL method achieves higher performance than the other methods across all scenarios. Therefore, these results indicate that learning the relationship between genotypes and Hill slope is more challenging than learning IC50 or IC80 independently. This may be due to statistical uncertainty in estimating the Hill slope from IC50 and IC80 values, which can also propagate into IIP estimates.

minor:

-"antibody-virus neutralization values" -- it would be helpful to be more precise in your initial description of the data, probably clarify catnap contains summary measures taken from in vitro TZMbl assays, and then define your measure of "sensitivity" as IC50, a summary statistic based on modeling titration curves

We agree and have now clarified that CATNAP data is obtained from in vitro TZM-bl assays. We have also noted that our typical metric for sensitivity will be IC50:

> Here, we will primarily use the half-maximal inhibitory concentration (IC50)—a standard summary statistic obtained by fitting antibody titration curves to estimate the antibody concentration required to achieve 50% neutralization—as our metric of virus “sensitivity” to antibodies.

-A large caveat we also addressed in that paper mentioned above is that the in vitro IC50 is far lower than the observed in vivo potency (ie bnAbs work much worse in vivo than they appear to in vitro) which might also be good to note, I unfortunately don't know at all if this scaling factor should be the same between bnAbs

Indeed, this is true and the complex relationship between in vitro neutralization and in vivo efficacy is an important caveat. We therefore added text to the Discussion clarifying that IC50 values are derived from in vitro assays and that absolute IC50 values should not be interpreted as direct measures of in vivo potency, which may differ across antibodies.

> It is also important to note that the IC50 values that we estimate are based on in vitro data sets using target cells that artificially express high levels of CD4 receptor. Thus, this measurement does not reflect in vivo potency directly, but rather an indirect, relative measure of neutralization susceptibility[30,42]. Absolute IC50 values therefore should not be interpreted as direct predictors of in vivo efficacy, and the relationship between in vitro neutralization and in vivo protection may vary across antibodies. Our approach is intended to compare relative sensitivity across viral variants and antibodies within the same experimental framework, rather than to infer absolute in vivo potency.

fig1a) I guess you don't need to redo all your work but from an interpretability pov it would be nice to show log10(IC50) so someone can naturally see 0.1, 1, 10 etc

Thank you for this suggestion, we have now updated this figure to show IC50 values as log10.

fig1c) isn't predicted usually on the x-axis for this type of diagram?

We have now updated this figure and other related figures (**Fig. 8c and Supplementary Fig. 5b**), comparing predicted-true values accordingly.

-is R really the right measure to assess prediction score? perhaps a concordance correlation coefficient and showing y=x and the slope of the best line may be more relevant and illuminating for bias

To address this point, we evaluated additional performance metrics beyond Pearson's correlation, including concordance correlation coefficient (CCC), regression slope, Spearman's rank correlation coefficient, and mean squared error (presented in a new **Fig. 3**). These complementary measures assess both agreement and potential bias between predicted and observed values. Across datasets and data fractions, these metrics yield conclusions consistent with those based on correlation alone, and CCC and slope in particular confirm improved agreement for the GNL method.

fig3) what are the error bars? should be mentioned in legend

We have now clarified the meaning of the error bars in the **Fig. 3** legend (now **Fig. 6** in the revised version of the paper). These error bars represent 95% confidence intervals based on a random selection of viruses used for cross-validation.

-"bnAbs" doesn't show up until page 6, not clear throughout which are bnAbs and which aren't

Thank you for pointing out this ambiguity. We have clarified throughout the manuscript which analyses involve broadly neutralizing antibodies (bnAbs) and which involve a broader set of antibodies.

In particular, our primary analyses using the CATNAP database include a diverse collection of antibodies, not all of which meet any single definition of a bnAb. We now explicitly state this when CATNAP is introduced and use the term "antibodies"

generically unless analyses are restricted to bnAbs. For the comparisons with SLAPNAP, we used a subset of bnAbs that they had selected for their own tests of performance. We have clarified this distinction in the corresponding section and revised the text to avoid referring to antibodies as bnAbs when the analysis is more general. For the within-host HIV-1 data, antibody names are summarized in the table in Supplementary Information, with their classification.

-did you ever explore whether certain bnAbs neutraliation is better predicted by looking only at partial sequences relevant for that binding site?

We did not restrict the sequence representation to specific Env regions for individual bnAbs. However, analysis of the learned model parameters shows that mutations at biologically relevant sites tend to carry larger weights and contribute disproportionately to predicted sensitivity, depending on antibody class. For example, sites 332 and 334, known to be critical for V3-glycan bnAbs, exhibit consistently higher weight magnitudes in models trained on V3-glycan antibody data. We present these results in the new **Interpretability of model parameters** section (new **Fig. 5** and **Supplementary Fig. 12, 13**, as well as **Supplementary Table 2**; see also the response to Reviewer 2, comment 1 below).

-I wondered how much error was introduced due to "resistance" saturation level. It might be useful to set all the saturated IC50s to be the same (e.g. 50µg/mL) and not worry about 20 vs 50 etc in your predictions

Thank you for this suggestion. We investigated the effect of using a fixed ceiling value (e.g., 50 µg/mL) for all saturated IC50 measurements. Unfortunately, this approach consistently reduced prediction accuracy across methods and data fractions. We therefore retained neutralization values as given in the data and noted this finding in the Supplementary Information:

> Since saturated IC50 values can vary across antibodies, we tested the effect of imposing a fixed ceiling of 50 µg/mL. This consistently resulted in lower accuracy across methods. Therefore, we retained neutralization values as given in each data set.

-refs have lots of (bibtex?) lower-case errors

Thank you for pointing this out. We have now corrected these errors.

Reviewer 2
This is a well-written manuscript that advances the problem of computational prediction of antibody neutralization of pathogens. The models are well-reasoned and rigorously tested, and comparisons to other state-of-the-art methods are fairly handled.

Major comments:

1) It is impressive that encoding viral sequences that the authors employ can predict held-out data well. However, we know from structure-function studies several critical motifs required for neutralization of specific bNAbs, and it is not clear if the reduced vector space for viral sequence encoding is sensitive enough to such mutations. For example, V3 glycan bNAbs typically require N332-glycan (not all, but most), and V2 apex bNAbs can be knocked-out by negatively charged amino acids at sites 169 and/or 171. Have the authors explored datasets of such mutants to test how well or not their models can predict the impact of these resistance mutations?

Such knockout mutations are also important in the clinical setting of passively transferred bNAb(s) to viremic participants – where such mutations can be selected for in vivo. I strongly recommend the authors to explore such clinical datasets to test if their methods can predict such resistance development due to a few Env mutations that were strongly selected for by the passively transferred bNAbs. If successful, this will also demonstrate the applicability of the authors' algorithm to this clinically relevant setting.

Thank you for this suggestion. To address whether the reduced sequence representation captures known resistance mutations, we analyzed mutation-specific contributions inferred by the GNL model. In the revised manuscript, we substantially expanded our interpretability analysis to address this concern directly.

Rather than focusing only on a small number of canonical resistance sites, we now systematically analyze mutation-specific effective weight parameters inferred from the trained GNL models. These effective weights quantify the contribution of individual amino acid substitutions to predicted neutralization sensitivity. Using this framework, we identify mutations with large effects that are shared across antibodies within the same antibody class, defined by the binding region.

This expanded analysis reveals that many of the highest-impact mutations correspond to known resistance-associated or sensitivity-associated sites for multiple antibody classes. For example, glycan-related mutations dominate the highest-impact set for V3-glycan antibodies, including mutations at sites 332 and 334 (note that we now explicitly model PNGSs — see response below). Mutations associated with other epitope classes sometimes show context-dependent effects consistent with prior experimental studies. These results are presented in the revised **Interpretability of model parameters** section, with corresponding figures (**Fig. 5** and **Supplementary Figs. 12–13**) and a summary table (**Supplementary Table 2**).

Together, these findings demonstrate that, despite operating in a reduced-dimensional sequence space, our GNL framework remains sensitive to biologically meaningful resistance mutations and recovers known structure-function relationships without relying on explicit structural annotations.

We agree also that resistance mutations selected under passive bnAb transfer in vivo represent an important and clinically relevant test case. While we have not explicitly modeled in vivo selection dynamics or longitudinal resistance emergence under bnAb therapy, the analysis described above shows that our approach is sensitive to large-effect Env mutations that are known to confer resistance across multiple bnAb classes. We would be interested to explore resistance evolution in future work, which should also consider important factors such as mutational accessibility (including, for example, the number of mutations needed to produce a specific resistance mutation given the observed virus population within an individual). We have discussed this limitation in the Discussion:

> It is also important to note that the IC50 values that we estimate are based on in vitro data sets using target cells that artificially express high levels of CD4 receptor. Thus, this measurement does not reflect in vivo potency directly, but rather an indirect, relative measure of neutralization susceptibility30,42. Absolute IC50 values therefore should not be interpreted as direct predictors of in vivo efficacy, and the relationship between in vitro neutralization and in vivo protection may vary across antibodies. Our approach is intended to compare relative sensitivity across viral variants and antibodies within the same experimental framework, rather than to infer absolute in vivo potency. Related to this point, our approach does not model virus sequence evolution, and thus GNL alone would not be sufficient to predict which antibody resistance mutations are most likely to evolve in an individual.

2) I recommend the authors to explore two potential avenues for biologically relevant characterization of viral sequence features, and test if these might improve the training-data fits and ultimately the prediction accuracy of their models:
i) Are the hypervariable loops of HIV Env treated any differently than the rest of the Env? It is well-known that these loops evolve rapidly by insertions/deletions in addition to point mutations; and this length and sequence variation implies that they cannot be meaningfully aligned. Therefore if the alignments were done differently in these regions, could they impact the encoding of the viral space which could then impact the neutralization predictions? Bette Korber et al. introduced alignment-free characteristics of length, net charge and total number of glycans to characterize these hypervariable loops and showed that each bNAb class was significantly impacted by one or more of these features (https://pubmed.ncbi.nlm.nih.gov/30629920/). It is not clear to me if use of numeric variables is amenable to the encoding strategy of the authors, but if possible, this might be interesting to test to see if training fits and prediction accuracy improve.

We agree that hypervariable Env loops pose a challenge for standard sequence alignment and that alignment-free features have been shown to be biologically relevant for bnAb sensitivity. In the revised manuscript, we therefore explicitly tested whether incorporating alignment-free features improves model performance.

Specifically, we added a new section, **Integration of alignment-free sequence features**, in which we integrated features describing loop length, net charge, and the number of potential N-linked glycosylation sites (PNGSs) in the V1-V5 regions into the GNL framework (see associated results in **Fig. 3** and **Supplementary Figs. 7-9**). These features were combined with the projected sequence representation and evaluated using multiple accuracy metrics, including Pearson's R, Spearman's rho, regression slope, concordance correlation coefficient, and MSE.

Across datasets and data fractions, we find that incorporating alignment-free features leads to only modest changes in overall prediction accuracy, with no systematic improvement for IC50 prediction, though slight gains are observed in some settings (e.g., Hill slope). These results suggest that, for the present data, standard sequence alignments are largely sufficient to learn genotype-neutralization relationships. However, alignment-free features may still provide complementary biological interpretability and prove useful in other contexts. Our findings are consistent with prior work highlighting the biological relevance of these features, while indicating that their predictive contribution may vary based on the source data set, predictive model, and the specific neutralization metric considered.

ii) In the "one-hot" encoding of viral sequences, the authors use an alphabet of 21 letters (20 amino acids and a gap). This alphabet does not include potential N-linked glycan sites (N-X-S or N-X-T, where X is any aa except Pro, PNGS). Virtually each known HIV bNAb has to interact with glycans, and often glycan gain/loss mutations (e.g. 332-NIS-334 to 332-NIN-334 for V3 glycan bNAbs) are important, even if the parental Asn site is not mutating. In contrast to above, the addition of one more letter in the alphabet ('O' for Asn in PNGS) should be straightforward to add to the already available encodings.

This is true, and we agree that glycan gain/loss/shift can be very important. We have therefore extended our model to include the 'O' symbol for PNGS. This modification allows the model to distinguish glycan-related mutations from other amino acid substitutions, including cases where glycan gain or loss occurs without mutation of the Asn itself.

In line with the reviewer's suggestion above, we also noted significant contributions from the mutations O332, N332, O334, and S334 for V3-glycan-binding antibodies. These mutations rank top 9.5, 411.5, 42.0, and 8.5 on average across 110 V3-glycan antibodies, corresponding to the top 0.11%, 4.97%, 0.51% and 0.10% of mutations, respectively. The corresponding p-values for inclusion within the top 1 percent across all observed mutations and V3-glycan antibodies are: $10^{-105.6}$, $10^{-31.9}$, $10^{-80.3}$, and $10^{-116.9}$. While PNGSs did not substantially improve model predictions, as described above, they do allow us to more precisely evaluate the predicted effects of glycans on neutralization in contexts such as this one.

Minor comments:

1) I recommend the authors to report significance using statistical tests (e.g. non-parametric tests) while reporting correlation coefficients between observed and predicted neutralization titers.

We agree that relying on a single correlation metric can be insufficient to fully assess prediction performance. In the revised manuscript, we therefore expanded our evaluation to include multiple complementary statistical measures, including Spearman's rank correlation coefficient (a non-parametric metric), regression slope, concordance correlation coefficient (CCC), and mean squared error (MSE), alongside Pearson's R.

These metrics assess different aspects of predictive accuracy, including rank ordering, absolute agreement, and bias. Across datasets and experimental conditions, the conclusions are consistent across metrics, and the additional measures provide a more complete and robust assessment of model performance (**Fig. 3**; **Supplementary Figs. 10–11**).

Regarding statistical significance, due to the large number of antibody-virus neutralization measurements in CATNAP (>81,000), all statistical associations between the predicted and true neutralization values for this data set are highly significant ($p < 10^{-100}$), even when most of the data is used for training. We have now added a line in the caption of **Figure 1** to clarify this point. Although the intrahost data from subject CH505 involve a much smaller number of observations than the CATNAP dataset (<2,400), we still observe a substantial level of statistical significance when predicting the sensitivity of novel viruses using 141 data points ($p < 10^{-11.8}$). When predicting randomly withheld sensitivities, the statistical significance is even stronger ($p < 10^{-90.9}$). We have clarified these analyses in the captions of **Figures 8** and **Supplementary Figure 6**.

2) I could not readily find the names of the antibodies used for example in the CH505 dataset. I urge the authors to provide summary tables in the supplement to quickly guide the readers about the makeup of the datasets used.

We have now included a brief description of the antibodies CH103 and CH235, isolated from the subject CH505 in the main section. Additionally, we included a table summarizing the data sources used in this study, including neutralization values measured with antibodies CH103 and CH235, derived from subject CH505.

Reviewer 3

This paper is an excellent contribution to the field of sequence based bnAb neutralization prediction. As there is a great interest in using anti-HIV antibodies for therapy and prevention this type of prediction algorithm is of great use to the field. My major comment is regarding your use of CV-R^2 over CV-RSME to assess the accuracy of your predictions and comparisons with SLAPNAP and Einav et al. methods. Both measures may be valuable but there

are cases where one might prefer a method with better RMSE. Consider additional reporting for comparison of the methods using RMSE.

Thank you for this comment — we agree that additional metrics of performance beyond Pearson correlations (including MSE) can be important. In the revised version of the manuscript, we have therefore reported additional statistics including the mean squared error, Spearman's rank correlation coefficient, regression slope, and concordance correlation coefficient. These metrics are reported for IC50 predictions in the main text (**Fig. 3**) and extended to additional measures in the Supplementary Information (**Supplementary Figs. 7-11**).

We were unfortunately unable to directly compute CV-RMSE from SLAPNAP due to data export issues (briefly, while we could export SLAPNAP fits to an entire data set and some pre-computed summary statistics, we could not output tests from individual cross validation runs). However, SLAPNAP provides cross-validation Spearman's rho values, which we have now reported in **Supplementary Fig. 5**.

Minor comments:
1. **In the abstract you state, "GNL can predict neutralization values for viruses with no observed data, an essential capability for evaluating novel viral strains." In what context are you evaluating novel viral strains? It seems like your method is best applied to characterizing the expected coverage or potency of a given bnAb or bnAb regimen applied to a specific population with a well characterized viral population.**

Thank you for this clarification. By "novel viral strains," our intention was to refer to viral sequences for which no neutralization measurements are available, rather than completely new/distinct ones. We agree that it would be most natural to apply our approach to estimate antibody coverage/potency within a population with well-characterized viral genetic diversity. To avoid ambiguity, we have now revised the Abstract to clarify this intended context and to emphasize prediction for sequences without observed neutralization data, rather than implying extrapolation well beyond the context of the training data:

> GNL can predict neutralization values for viral sequences without observed neutralization measurements, an important capability for assessing antibody coverage in populations whose viral diversity is genetically characterized.

To further clarify this point, we have also made it clear in the Discussion that

> …as with any sequence-based prediction approach, our method is limited by the variants observed in the training data; rare or unobserved variants, which can be clinically important drivers of escape, might not be explicitly captured.

2. In the introduction it seems relevant to include the AMP human anti-HIV bnAb efficacy trials.

Yes, we agree that this is relevant to the current work. We have now revised the Introduction to mention AMP along with other antibody-based therapies:

> Passive immunization with broadly neutralizing antibodies (bnAbs) is a promising treatment to prevent infection or control viral progression, and its clinical relevance has been demonstrated in large-scale human studies such as antibody-mediated prevention (AMP) trials[8]. These efforts highlight the need for methods to systematically characterize how viral genetic variation affects antibody susceptibility or resistance.

3. In the section on "Grouping similar antibodies" the subscript for the similarity matrix ω appears to have two levels. I think it should be one.

Thank you for pointing this out, we have now corrected the subscript.

4. In this same section the sentence "is defined as a product of the angular similarity of their neutralization vectors against the same viruses and the fraction of neutralization values that are shared…". What is your rationale for including the "fraction of neutralization values that are shared" as part of the weight? It would help to have some intuition for why you would include this factor. It seems counter intuitive to increase similarity just because the antibodies have both been tested against the same viruses.

We thank the reviewer for raising this point and agree that the intuition for including a shared-fraction term was unclear in the earlier description. In fact, in the current version of the manuscript, we now define antibody similarity solely by the angular (cosine) similarity of neutralization vectors restricted to shared virus measurements (Eq. 5).

In our first analyses, we explored different approaches to downweight similarity estimates based on very small numbers of shared measurements, which was motivated by concerns about statistical noise. However, upon further testing we found that this scaling actually had very little effect on the accuracy of our predictions, and as noted above, could cause confusion. Therefore, we have now removed this factor, and all results presented in this study use cosine similarity alone. We have revised the text in both the main manuscript and Methods to reflect this choice and to avoid confusion.

5. In the section "Extracting sequence features and learning neutralization values", you describe "projected each viral sequence onto the eigenvectors". I assume these are aligned sequences. Consider specifying that the sequences have been aligned.

We agree that this is an important detail, and we have now included a sentence at the beginning of the section that states, “First, we align the ensemble of viral sequences (below, we simply refer to aligned sequences as sequences).”

6. In Figure 1 panel d, how are the bars (uncertainty) around each point defined?

These error bars are 95% confidence intervals based on random partitions of the neutralization data into training and testing subsets. We have now clarified this in the caption.

7. In Figure 1 panels c and d, which value of rho was used? It would help to specify the value in the figure caption.

These rank values are estimated based on our criterion of selecting “optimal” rank values. We have now added a description to the caption: “The rank of the low-rank approximation is selected as the smallest value for which the cumulative fraction of variance explained by the retained eigenmodes exceeds 95% (see Methods).”

8. **In Figure 2 panel e, the GNL the curve is surprisingly flat. Do you believe this result?**

Thank you for this question. We believe the relatively flat behavior of the GNL curve in **Fig. 2e** is expected and reflects a key feature of the method. In GNL, predictive information is shared across antibodies with similar neutralization profiles, allowing antibodies with limited training data to benefit from related, more extensively characterized antibodies. As a result, GNL performance is less sensitive to the number of measurements available for any single antibody and degrades less severely for antibodies with fewer measurements.

In contrast, methods that treat antibodies independently rely entirely on per-antibody data and therefore show a stronger dependence on the number of training measurements. We interpret the flatter GNL curve as increased robustness to data sparsity, rather than as a lack of benefit from additional measurements.

9. In Figure 2, panels i-j, please add % to the x-axis label.

We have corrected the figures accordingly.

10. In Figure 4, panels b and c appear to be swapped.

We apologize for any confusion caused by the figure title. Figure 4, panels b and c,

as well as their titles, are presented as intended. In panel b, the model is tested on data from weeks 210 to 555, while data from weeks 28 to 140 are used for training. Similarly, in panel c, the model is trained on data from weeks 210 to 555 and tested on data from weeks 28 to 140. We have updated the figure to make these titles more explicit and clearer.

11. In Figure 5 panel c, it would help to show RMSE or at least acknowledge the negative bias in your predictions.

We have now included both the RMSE and Spearman's correlation values in the caption, as well as the bias toward smaller (predicted) IC50 values:

> GNL's (Einav et al.'s) Pearson's *R*, Spearman's rho, linear regression slope and MSE values are 0.55 (-0.02), 0.57 (-0.11), 1.53 (-0.07), and 11.05 (11.28), respectively. As indicated by the regression slope, the predicted values are systematically smaller than the true ones in this example.

12. In the section on "Comparison against alternative approaches for virus antibody neutralization prediction", you make the statement that SLAPNAP uses Bliss-Hill for combinations of antibodies. At this point in the manuscript it's not clear that your method has been applied to combinations of antibodies. From the discussion section it appears that you are not using the data in CATNAP on antibody combinations. It would be helpful to clarify.

Thank you for this point, we have now clarified that, in this paper, we do not consider predictions for combinations of antibodies.

In closing, we appreciate the opportunity to revise our manuscript, and we are grateful to the reviewers for their constructive comments, which have helped to strengthen the paper.

Sincerely,
John Barton
Department of Computational and Systems Biology
University of Pittsburgh School of Medicine
