# Supplementary material for: Predicting viral sensitivity to antibodies using genetic sequences and antibody similarities
Source: PLoS Comput Biol. 2026 Mar 23;22(3):e1014095. doi: 10.1371/journal.pcbi.1014095 (PMC13020759; doi:10.1371/journal.pcbi.1014095)
Supplement: Attachment — Submitted filename: PCOMPBIOL-D-25-01851_comments.docx [file pcbi.1014095.s020.docx]

This paper is an excellent contribution to the field of sequence based bnAb neutralization prediction. As there is a great interest in using anti-HIV antibodies for therapy and prevention this type of prediction algorithm is of great use to the field. My major comment is regarding your use of CV-R^2 over CV-RSME to assess the accuracy of your predictions and comparisons with SLAPNAP and Einav et al. methods. Both measures may be valuable but there are cases where one might prefer a method with better RMSE. Consider additional reporting for comparison of the methods using RMSE.

Minor comments:

1. In the abstract you state, “GNL can predict neutralization values for viruses with no observed data, an essential capability for evaluating novel viral strains.” In what context are you evaluating novel viral strains? It seems like your method is best applied to characterizing the expected coverage or potency of a given bnAb or bnAb regimen applied to a specific population with a well characterized viral population.
2. In the introduction it seems relevant to include the AMP human anti-HIV bnAb efficacy trials.
3. In the section on “Grouping similar antibodies” the subscript for the similarity matrix ω appears to have two levels. I think it should be one.
4. In this same section the sentence “is defined as a product of the angular similarity of their neutralization vectors against the same viruses and the fraction of neutralization values that are shared…”. What is your rationale for including the “fraction of neutralization values that are shared” as part of the weight? It would help to have some intuition for why you would include this factor. It seems counter intuitive to increase similarity just because the antibodies have both been tested against the same viruses.
5. In the section “Extracting sequence features and learning neutralization values”, you describe “projected each viral sequence onto the eigenvectors”. I assume these are aligned sequences. Consider specifying that the sequences have been aligned.
6. In Figure 1 panel d, how are the bars (uncertainty) around each point defined?
7. In Figure 1 panels c and d, which value of rho was used? It would help to specify the value in the figure caption.
8. In Figure 2 panel e, the GNL the curve is surprisingly flat. Do you believe this result?
9. In Figure 2, panels i-j, please add % to the x-axis label.
10. In Figure 4, panels b and c appear to be swapped.
11. In Figure 5 panel c, it would help to show RMSE or at least acknowledge the negative bias in your predictions.
12. In the section on “Comparison against alternative approaches for virus antibody neutralization prediction”, you make the statement that SLAPNAP uses Bliss-Hill for combinations of antibodies. At this point in the manuscript it’s not clear that your method has been applied to combinations of antibodies. From the discussion section it appears that you are not using the data in CATNAP on antibody combinations. It would be helpful to clarify.
